# Multiple *Mycobacterium abscessus O*-acetyltransferases influence glycopeptidolipid structure and colony morphotype

**DOI:** 10.1016/j.jbc.2023.104979

**Published:** 2023-06-28

**Authors:** Morgane Illouz, Louis-David Leclercq, Clara Dessenne, Graham Hatfull, Wassim Daher, Laurent Kremer, Yann Guérardel

**Affiliations:** 1 Centre National de la Recherche Scientifique UMR 9004, Institut de Recherche en Infectiologie de Montpellier (IRIM), Université de Montpellier, Montpellier, France; 2 UMR 8576 - UGSF - Unité de Glycobiologie Structurale et Fonctionnelle, CNRS, Université de Lille, Lille, France; 3 Department of Biological Sciences, University of Pittsburgh, Pittsburgh, Pennsylvania, USA; 4 INSERM, IRIM, Montpellier, France; 5 Institute for Glyco-Core Research (iGCORE), Gifu University, Gifu, Japan

**Keywords:** *Mycobacterium abscessus*, glycopeptidolipid, cell wall, acetyltransferase, macrophage, mass spectrometry

## Abstract

*Mycobacterium abscessus* causes severe lung infections. Clinical isolates can have either smooth (S) or rough (R) colony morphotypes; of these, S but not R variants have abundant cell wall glycopeptidolipids (GPL) consisting of a peptidolipid core substituted by a 6-deoxy-α-L-talose (6-dTal) and rhamnose residues. Deletion of *gtf1*, encoding the 6-dTal transferase, results in the S-to-R transition, mycobacterial cord formation, and increased virulence, underscoring the importance of 6-dTal in infection outcomes. However, since 6-dTal is di-*O*-acetylated, it is unclear whether the *gtf1* mutant phenotypes are related to the loss of the 6-dTal or the result of the absence of acetylation. Here, we addressed whether *M. abscessus atf1* and *atf2*, encoding two putative *O*-acetyltransferases located within the *gpl* biosynthetic locus, transfer acetyl groups to 6-dTal. We found deletion of *atf1* and/or *atf2* did not drastically alter the GPL acetylation profile, suggesting there are additional enzymes with redundant functions. We subsequently identified two paralogs of *atf1* and *atf2*, *MAB_1725c* and *MAB_3448*. While deletion of *MAB_1725c* and *MAB_3448* had no effect on GPL acetylation, the triple *atf1-atf2-MAB_1725c* mutant did not synthetize fully acetylated GPL, and the quadruple mutant was totally devoid of acetylated GPL. Moreover, both triple and quadruple mutants accumulated hyper-methylated GPL. Finally, we show deletion of *atf* genes resulted in subtle changes in colony morphology but had no effect on *M. abscessus* internalization by macrophages. Overall, these findings reveal the existence of functionally redundant *O*-acetyltransferases and suggest that *O*-acetylation influences the glycan moiety of GPL by deflecting biosynthetic flux in *M. abscessus.*

Infections caused by non-tuberculous mycobacteria (NTM), and particularly *Mycobacterium abscessus*, are globally on the rise and remain difficult to treat due to their intrinsic resistance levels to most antibiotic classes (1). *M. abscessus* is a fast-growing bacteria, increasingly acknowledged as an emerging human pathogen, responsible for skin, soft tissue (2), and lung infections, mostly in patients with underlying lung disorders, such as patients with cystic fibrosis (CF) (3, 4). *M. abscessus* displays either smooth (S) or rough (R) colony morphotypes to which distinct *in vitro* and *in vivo* phenotypes can be assigned. S variants are typified by the production of surface-associated glycopeptidolipids (GPL), which are lacking or produced at low levels in the R variants (5, 6, 7).

GPL are complex lipids (Fig. 1*A*) comprising a D-Phe-D-*allo*Thr-D-Ala-L-alaninol peptide core assembled by the action of two non-ribosomal peptide synthetases, Mps1 and Mps2, and acylated with a 3-hydroxy/methoxy C_24_–C_33_ fatty acid (8, 9, 10). It was recently shown that alaninol can be replaced by branched amino-alcohol valinol or leucinol (11). The resulting lipopeptide is glycosylated with 6-deoxy-α-L-talose (6-dTal) on the *allo*-Thr residue by the action of the glycosyltransferase Gtf1, while the alaninol is substituted by a α-L-rhamnose (Rha) by Gtf2, resulting in the production of the less-polar diglycosylated GPL molecules, designated GPL-2a. In addition to GPL-2a that contains a 3,4-di-*O*-acetylated 6-dTal and a 3,4-di-*O*-methylated or 2,3,4-tri-*O*-methylated Rha (8, 12, 13, 14), *M. abscessus* also produces a more polar GPL, designated GPL-3, through the addition of a 2,3,4-tri-hydroxylated Rha to the alaninol-linked 3,4-di-*O*-methyl Rha, a reaction catalyzed by glycosyltransferase Gtf3 (15). GPL-3 is structurally identical in *M. abscessus* and *Mycobacterium smegmatis* but is more abundant in *M. abscessus* (8). Once synthesized, GPL-2a and GPL-3 are translocated across the inner membrane by the MmpL4a/MmpL4b transporters (16, 17, 18, 19) and inserted into the outer leaflet of the mycomembrane where they are exposed to the surface of the bacilli (20).

**Figure 1:**
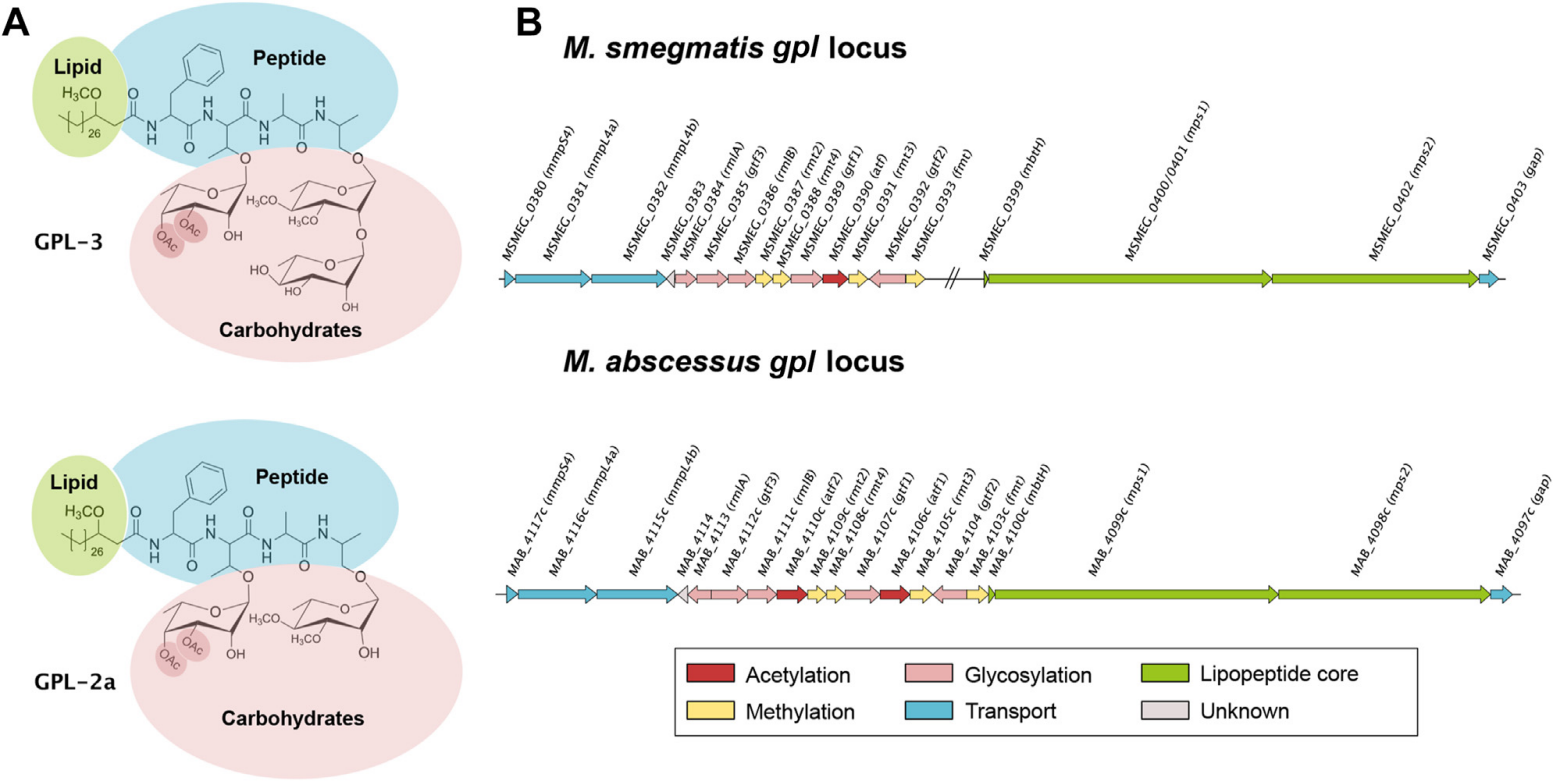
**Prediction of GPL acetyltransferases in *M*. *abscessus.****A*, the two major structures of GPL are schematized with a lipid chain (*green*), a peptide core (*blue*), and a glycan moiety (*pink*). Tri-glycosylated GPL, designated GPL-3, is composed of a 6-dTal and two Rha residues. Di-glycosylated GPL, designated GPL-2a, possesses only one Rha. The acetyl groups on the C3 and C4 positions of 6-dTal are highlighted by a *red circle*. Internal Rha residues are methylated in C3 and C4 positions. *B*, *M. smegmatis* and *M. abscessus gpl* loci encode enzymes for the synthesis, modification, and transport of GPL.

Numerous studies showed that the presence or loss of GPL influences susceptibility to antibiotics, sliding motility, biofilm formation (5, 7, 16, 21, 22, 23), bacterial surface hydrophobicity (20, 24), cord formation (5, 16, 25, 26), interaction with host macrophages (13, 27), and induction of pro-inflammatory responses (28). The role of S/R morphotypes to influence disease outcomes is supported by cellular and animal models, indicating increased pathogenesis of R forms relative to S forms (16, 26, 29, 30). In particular, in the zebrafish model of infection, the S-to-R transition is associated with increased bacterial loads, production of mycobacterial cords, abscess formation, and enhanced larval mortality (29). Understanding the molecular mechanisms behind the switch from S to R morphotype is of paramount importance and clinically relevant as evidenced by epidemiological surveys highlighting the predominance of R strains in patients with severe lung diseases and chronic colonization of the airways in CF patients (31, 32). Genomic and transcriptomic analyses originally revealed the presence of various insertions or deletions in the R strains, mostly found in *mps1*, *mps2*, and *mmpL4b* genes participating either in the biosynthesis or transport of GPL and responsible for the S-to-R transition in *M. abscessus* (33, 34, 35). A recent in-depth dissection study of the GPL glycome provided insights into the biological function of the different monosaccharides and demonstrated that deletion of either *gtf1* or *gtf2* (but not *gtf3*) resulted also in the S-to-R transition with enhanced cording and increased virulence in zebrafish embryos (15). These findings emphasize the importance of 6-dTal in infection outcomes and in the interaction with host macrophages. However, because 6-dTal is diacetylated, it is not known whether the phenotypes associated with *gtf1* deletion are a direct consequence of the absence of the 6-dTal or caused by the loss of the 6-dTal-substituted acetyl groups. In addition, whether the presence/absence of 6-dTal acetylation influences the GPL structure is not well understood. Thus, the identification of the *O*-acetyl transferases (Atf) and the contribution of *O*-acetylation to the overall GPL structure and biological function in *M. abscessus* remain to be established.

The aim of this study was to further advance our understanding of the genetic requirements for the biosynthesis and biological functions of the high-GPL-producing variants by focusing on the Atf-related enzymes involved in GPL *O*-acetylation. Bioinformatics and genetic studies were combined to identify and generate single and multiple deletion mutants lacking one to four *atf* genes and further characterized by functional complementation studies followed by detailed biochemical and structural lipid analyses. These mutants were also exploited to address the contribution of *O*-acetylation during the synthesis of GPL, their role in bacterial morphology and internalization of *M. abscessus* in human macrophages.

## Results

### Generation of *atf1* and *atf2* acetyltransferases deleted strains

While previous studies aimed at deciphering the contribution of the different moieties composing GPL (*O*-methylation of the lipid core (24) or glycosylation of the peptide backbone (15)) to the biological functions of this glycolipid, the importance of GPL acetylation on C3 and C4 positions of the 6-dTal of GPL-2a and GPL-3 (Fig. 1*A*) remains unsolved. The *M. abscessus gpl* biosynthetic locus encompasses two genes coding for putative acetyltransferases, referred to as *atf1 (MAB_4106c)* and *atf2 (MAB_4110c)* (8), contrasting with *M. smegmatis* which possesses a single *atf* gene *(MSMEG_0390)* (36) (Fig. 1*B*); all of these are predicted to be membrane-localized and contain ten putative transmembrane domains. To investigate the contribution and biological functions of Atf1 and Atf2 in GPL synthesis, *atf1* and *atf2* were deleted either individually or simultaneously in a high-GPL-producing *M. abscessus* S variant (CIP104536^T^; identical to ATCC19977), using an unmarked deletion system that allows multiple gene deletions in *M. abscessus* (37). In brief, the strategy involves double homologous recombination leading to the deletion of the *atf1* and/or *atf2* open reading frames from the *gpl* locus, based on the pUX1-*katG* suicide vector (Table S1) carrying a kanamycin resistance (*kan*^*R*^) cassette, a tdTomato fluorescence marker, and a *katG* cassette that confers susceptibility to isoniazid (INH) in *M. abscessus*. The DNA segments flanking *atf1* or *atf2* were cloned into pUX1-*katG,* yielding pUX1-*katG-atf1* and pUX1-*katG-atf2* (Fig. S1*A*). Transformants that integrated the plasmids into their specific loci *via* homologous recombination were selected for resistance to kanamycin and had visible red fluorescence. After a second homologous recombination event, loss of the plasmid resulted in the recovery of *Δatf1* (designated Δ1) and *Δatf2* (designated Δ2) non-fluorescent colonies that were INH-resistant and Kan-susceptible. PCR/sequencing of the progenitor and the deletion strains using primers listed in Table S2, confirmed the genotype of these *atf* mutants (Fig. S1*B*). Functional complementation of Δ1 and Δ2 was done through specific integration at the *attB* chromosomal site (38) of an intact copy of *atf1* and *atf2* with an HA-tag placed at the 3′-end and under the control of the *hsp60* promoter (Fig. S2*A*). Western blotting of the crude lysates using anti-HA antibodies was carried out by loading equal amounts of proteins (confirmed by the KasA protein internal control) (Fig. S2*B*). This revealed the presence of single bands, corresponding to Atf1-HA and Atf2-HA, although these proteins migrated slightly faster than their predicted sizes, validating their expression in the complemented Δ1 and Δ2 strains.

### Deletion of *atf1* and/or *atf2* does not suppress GPL acetylation

**Figure 2:**
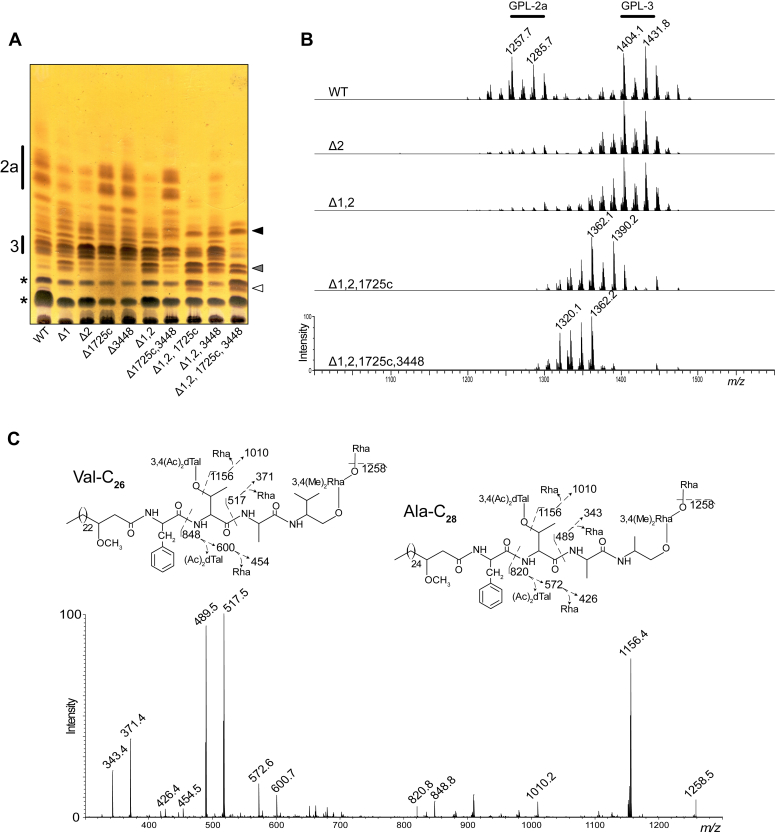
**The GPL profile varies according to deleted acetyltransferases.***A*, TLC analysis of the polar lipid fraction of wild-type (WT) and acetyltransferases mutant strains. Previously characterized GPL-2a and GPL-3 mobility (15, 24) are shown on the *left side* (2a and 3); newly observed accumulating GPL are marked on the *right side* as *white*, *grey*, and *black arrowheads*; diacyl-trehalose isomers (DAT) identified by NMR are highlighted with an *asterisk*. *B*, MALDI-MS spectra in positive mode of the polar lipid fraction of wild-type (WT) and selected acetyltransferases mutant strains. Previously characterized GPL-2a and GPL-3 (15, 24) MS signals are shown on *top* of the spectra. *C*, MS^2^ fragmentation spectrum of the selected parent ion at *m/z* 1404. Fragmentation patterns are illustrated for two GPL-3 isomers with alaninol and C_28_ lipid (Ala-C_28_) or valinol and C_26_ lipid (Val-C_26_). Each one produces the same fragment ions when di-*O*-acetylated 6-dTal and/or Rha are lost, but Y fragments ions are shifted by 28 a.m.u. along the peptidic backbone.

To qualitatively analyze the impact of *atf1* and/or *atf2* deletion, we analyzed the GPL from WT, Δ1, Δ2, and Δ1,2 strains (Table S3). GPL was prepared by sequential extraction of apolar and polar lipid fractions and analyzed by thin-layer chromatography (TLC). Apolar fractions all exhibited similar profiles (Fig. S3*A*) and were not further studied. In contrast, the GPL profiles in polar fractions of Δ1, Δ2, and Δ1,2 differed from the parental S strain (Fig. 2*A*), whereas other glycolipids are expressed at similar levels (Fig. S3*B*). In particular, the band intensity of GPL-2a significantly decreased in Δ1, Δ2, and Δ1,2 compared to WT. TLC analysis also showed an increased intensity for a set of bands with slower mobility, potentially more polar than GPL-3, in all three mutants; this was more pronounced in Δ1,2 (Fig. 2*A*, gray arrowhead).

The MALDI-MS spectrum of polar fraction from the WT strain reveals the presence of a complex set of GPL dominated by multiple isomers of GPL-3 (major signals at *m/z* 1404 and 1432) and GPL-2a (major signals at *m/z* 1258 and 1286) (Fig. 2*B*), as reported previously (13, 15, 24, 39). The presence of di-*O*-acetylated GPL-3 was confirmed in all four samples by MS/MS analysis of the parent *m/z* 1404 ion that produced the diagnostic fragment ion M-248 at *m/z* 1156, typifying the loss of 3,4 di-*O*-acetylated 6-dTal (Fig. 2*C*). Detailed analysis of the fragmentation spectra showed several fragment ions with 28 a.m.u. differences that originate from either the variation in the length of the lipid chain (C_26_ or C_28_) or the variation in the peptide sequence (alaninol or valinol), generating intrinsic heterogeneity of GPL-3, designated Ala-C_28_ or Val-C_26_ (Fig. 2*C*). It should be noted that the internal Rha residue linked to the amino alcohol is 3,4 di-*O*-methylated. MS/MS analysis of the signal at *m/z* 1404 in all four strains resulted in the same fragmentation spectrum, demonstrating that Δ1 and Δ2 synthesize similar di-*O*-acetylated GPL-3 (Fig. S4*A*). Comparable observations were made with the isomeric structure of GPL-3 at *m/z* 1432 (Fig. S4*B*). As for GPL-3, GPL-2a was observed in all four strains with similar structures. However, in accordance with the TLC analysis, the intensity of GPL-2a MS signals at *m/z* 1258 and 1286 was strongly decreased in Δ2 and Δ1,2 when compared to the GPL-3 signals at *m/z* 1404 and 1432 (Fig. 2*B*) but not in Δ1 (Fig. S5). Importantly, the complementation of Δ1 and Δ2 restored the parental GPL profile (Fig. S3*C*). At this stage of the study, we could not identify the product accumulating in Δ1, Δ2 and Δ1,2 (Fig. 2*A*, grey arrowhead) due to spectral overlap but this was later identified as mono*-O-*acetylated GPL-3 (see below).

Together, these observations show that the single and double disruptions of *atf1* and *atf2* induced moderate under-acetylation of GPL-3 associated with an unexpected reduction of GPL-2a amounts compared to GPL-3, which was more pronounced in the absence of *atf2*.

### Bioinformatic analyses identify two *atf* paralogues in *M. abscessus*

**Figure 3:**
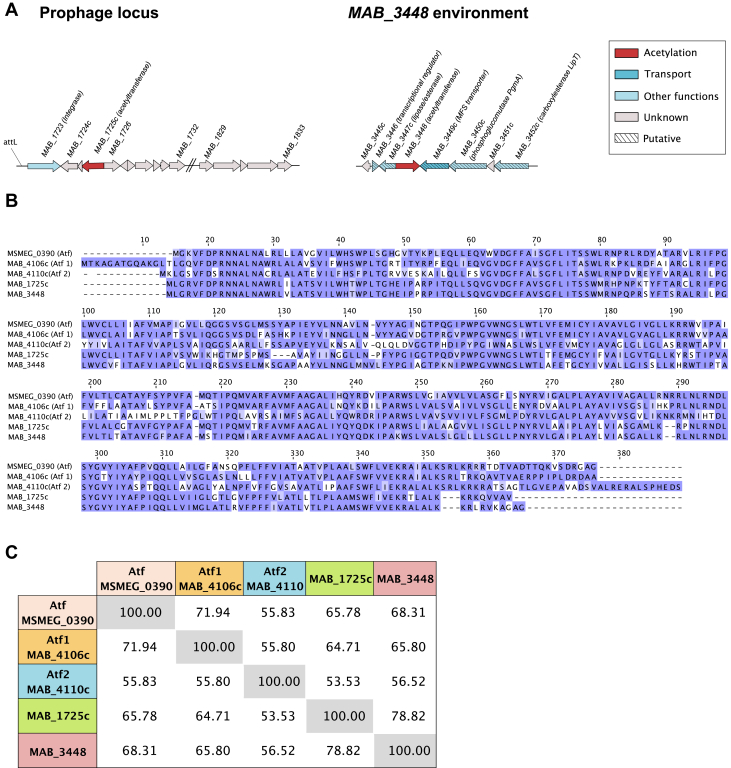
**Paralogues of *atf1* and *atf2* are present in the *M. abscessus* genome.***A*, genomic organization around the two putative acetyltransferases encoding genes, *MAB_1725c* and *MAB_3448*. *B*, alignment of the protein sequence of the *M. smegmatis* GPL acetyltransferase (Atf) with the putative *M. abscessus* GPL acetyltransferases. *C*, cross-tabulation of the percentage of identity between the five acetyltransferases.

The fact that the acetylation profile was incompletely modified in Δ1,2 raised the possibility of additional enzymes with redundant functions, encoded by genes outside of the *gpl* locus. BLAST analyses using either Atf1 or Atf2 as queries identified two putative acetyltransferase candidates, encoded by *MAB_1725c* and *MAB_3448*, respectively. Interestingly, *MAB_1725c* is part of a previously identified prophage (designated prophiATCC19977-1) in *M. abscessus* ATCC19977 (Fig. 3*A*) and can spontaneously induce to give viable phage particles (40, 41). The prophage is integrated into *attB-5* overlapping a host *tRNA*^*Met*^ gene and has 113 predicted coding sequences (35, 40, 41). In contrast, *MAB_3448* belongs to a cluster of genes coding for proteins with unknown function or expressing lipolytic activity, such as MAB_3447c or MAB_3452c (Fig. 3*A*). Multiple amino acid alignments show the high sequence conservation between all four proteins (Fig. 3*B*). MAB_1725c shares 65% and 53% identity with Atf1 and Atf2 while MAB_3448 shares 66% and 57% identity with Atf1 and Atf2, respectively. MAB_1725c and MAB_3448 share 79% identity (Fig. 3*C*). This high sequence conservation suggests these enzymes may be functionally redundant. Like Atf1 and Atf2, MAB_1725c and MAB_3448 are predicted to be membrane localized.

### Loss of *atf1*, *atf2*, *MAB_1725c*, and *MAB_3448* severely impact the GPL composition

To investigate the potential contribution of *MAB_1725c* and *MAB_3448* to GPL acetylation, these genes were individually or simultaneously deleted in *M. abscessus* using the genetic approach employed to generate Δ1 and Δ2, leading to Δ1725c, Δ3448, and Δ1725c,3448 (Table S3 and Fig. S1*C*). The deletions were confirmed by PCR/sequencing (Fig. S1*D* and Table S2), and the mutants were subjected to lipid analyses. TLC (Fig. 2*A*) as well as MALDI-MS profiling (Fig. S5) of Δ1725c, Δ3448, and Δ1725c,3448 closely mirror the WT pattern, demonstrating that *MAB_1725c* and *MAB_3448* are not essential for acetylation of GPL in a WT strain. Next, we tested the possibility that these enzymes express redundant activities with Atf1 and/or Atf2 by comparing the GPL profiles of Δ1, Δ2, Δ1,2 with Δ1,2,1725c and Δ1,2,3448 as well as in the quadruple mutant (Δ1,2,1725c,3448) lacking the four genes. Δ1,2,3448 showed by TLC and MALDI-MS analyses a GPL profile comparable to Δ1,2 (Figs. 2*A* and S5). Conversely, Δ1,2,1725c exhibited a different profile characterized by a strong reduction of GPL-2a and GPL-3 but with a concomitant increase in the intensity of the lower Rf bands (Fig. 2*A*). Furthermore, two intense sets of bands appeared just below the GPL-3 (Fig. 2*A*, white and gray arrowheads) and one between GPL-3 and GPL-2a (Fig. 2*A*, black arrowhead), suggesting that biosynthetic intermediates accumulate concomitantly to the disappearance of the final products. A similar trend was observed in Δ1,2,1725c,3448, culminating with the total disappearance of GPL-2a and GPL-3 bands (Fig. 2*A*). The polar glycolipid content was also assessed by TLC using other developing solvents, but no significant changes outside GPLs were observed (Fig. S3, *D* and *E*). Consistently, MALDI-MS spectra of Δ1,2,1725c and Δ1,2,1725c,3448 displayed very different profiles to the WT strain, characterized by the strong reduction of GPL-3 signals at *m/z* 1404 and 1432 in the former and their complete disappearance in the latter (Fig. 2*B*). The MALDI-MS spectrum of Δ1,2,1725c showed a new cluster of signals dominated by a pair of ions at *m/z* 1362 and 1390, tentatively identified as mono-*O*-acetylated GPL-3 based on a 42 a.m.u reduction compared to GPL-3 signals at *m/z* 1404 and 1432. A cluster of ions from *m/z* 1320 to 1362 was identified in Δ1,2,1725c,3448 (Fig. 2*B*), requiring further investigation.

### *M. abscessus* lacking *atf1*, *atf2*, *MAB_1725c*, and *MAB_3448* produce non-acetylated and hyper-methylated GPL

**Figure 4:**
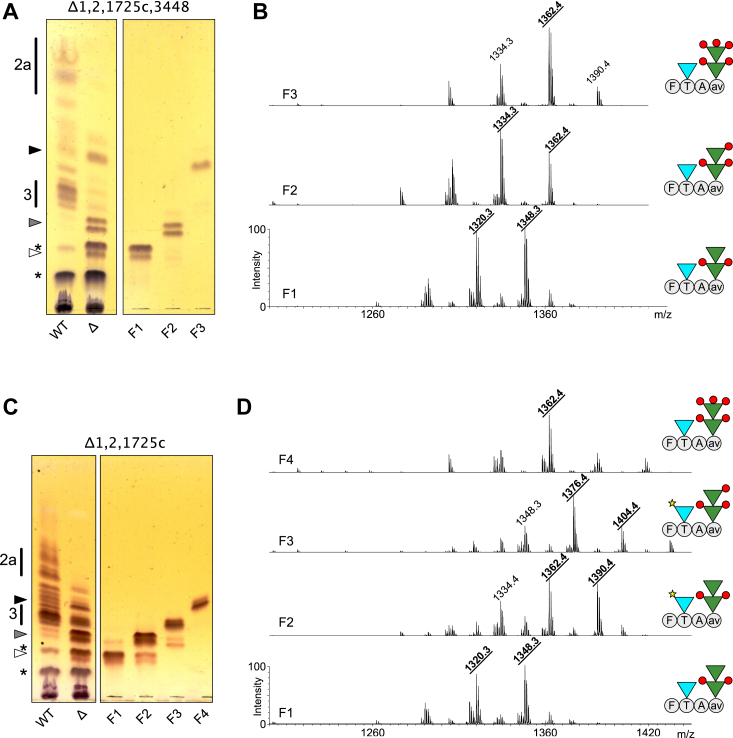
**GPL from *M. abscessus* Δ1,2,1725c and Δ1,2,1725c,3448 are under-acetylated and hyper-methylated.***A* and *B*, GPL profile of Δ1,2,1725c,3448; (*C* and *D*) GPL profile of Δ1,2,1725c. *A* and *C*, TLC analyses of Δ1,2,1725c and Δ1,2,1725c,3448 before and after separation in three (F1–F3) or four (F1–F4) fractions, respectively, as compared with wild-type (WT). *White*, *grey*, and *black arrowheads* correspond to the accumulating products observed on TLC plates of total polar fractions. *B* and *D*, MALDI-MS spectra of Δ1,2,1725c,3448-F1 to F3 and Δ1,2,1725c-F1 to F4 following the separation of total polar fractions. Increasing *m/z* values are associated with additional acetyl group (*yellow star*) on the 6-dTal and *O*-methyl groups (*red circles*) on the terminal Rha. GPL schemes are drawn according to single-letter amino-acids code and SNFG nomenclature.

To explore the complex nature of the structural modifications observed in the different mutant strains, we first focused on the quadruple mutant. Indeed, deletion of all four *atf* genes induced substantial biochemical alterations, resulting in the simplest GPL profile of all studied mutants, characterized by the accumulation of three lipids, as observed on the TLC (Fig. 2*A*, black, gray, and white arrowheads). The structures of these three components were individually determined in order to provide the basis for analyzing the more complex triple mutant. First, the polar lipid fraction isolated from the quadruple mutant was separated by preparative TLC into three fractions, referred to as Δ1,2,1725c,3448-F1, Δ1,2,1725c,3448-F2 and Δ1,2,1725c,3448-F3 (Fig. 4*A*). Δ1,2,1725c,3448-F1, which co-elutes with di-acyl trehalose (DAT), showed two major ions at *m/z* 1320 and 1348 on the MALDI-MS spectrum (Fig. 4*B*), tentatively attributed to non-acetylated GPL-3 based on the 84 a.m.u decrease from ions *m/z* 1404 and 1432. MS^2^ fragmentation confirmed that the GPL signal at *m/z* 1320 was made of two GPL-3 isomers Ala-C_28_ and Val-C_26_, substituted by non-acetylated 6-dTal and Rha residues, owing to the observation of signals M-6dTal at *m/z* 1156 and M-Rha at *m/z* 1174, respectively (Fig. S6*A*). The GPL signal at *m/z* 1348 was identified as a GPL-3 isomer Val-C_28_ substituted by identical monosaccharides (Fig. S6*B*). Δ1,2,1725c,3448-F2 comprises two bands with intermediate Rf between Δ1,2,1725c,3448-F1 and GPL-3 (Fig. 4*A*). MALDI-MS analysis showed a group of three intense ions dominated by the ion at *m/z* 1334 (Fig. 4*B*). MS^2^ fragmentation generated a signal at *m/z* 1170 indicative of the loss of non-acetylated 6-dTal as well as a M-160 ion at *m/z* 1174, indicative of the loss of terminal mono-*O*-methyl Rha from non-acetylated GPL-3 isomers Ala-C_28_ and Val-C_26_ (Fig. S7*A*). Parent ion at *m/z* 1362 displayed a similar MS^2^ pattern, highlighting the loss of non-acetylated 6-dTal and mono-*O*-methyl Rha, which typifies a GPL-3 Val-C_28_ form (Fig. S7*B*). It should be noted that tri-*O*-methylated GPL-3 with identical signal at *m/z* 1334 was previously reported in *M. smegmatis* (42). Finally, Δ1,2,1725c,3448-F3 was characterized as a single high mobility band on TLC, associated to a set of major ions at *m/z* 1334, 1362 and 1390 on MALDI-MS (Fig. 4, *A* and *B*). The 28 a.m.u increase in the parent ion over Δ1,2,1725c,3448-F2, coupled with the Y2-fragment ion generated by MS/MS at *m/z* 531, suggested the presence of two additional *O*-methyl groups. These were located on the terminal Rha owing to the MS^3^ fragmentation of the Y2 ion at *m/z* 531 that generated a secondary fragment M-188 ion at *m/z* 343, confirming the presence of a terminal tri-*O*-methyl Rha on GPL-3 Ala-C_28_ (Fig. S8*A*). Similar MS^2^ fragmentation patterns were observed for parent ion at *m/z* 1390, which typifies GPL-3 Ala-C_30_ and Val-C_28_ isobars with terminal tri-*O*-methyl Rha (Fig. S8*B*). The presence of tri-*O*-methyl Rha was then confirmed by GC/MS analysis as demonstrated below.

Together, these data demonstrate that the quadruple mutant Δ1,2,1725c,3448 completely lost the ability to synthesize acetylated GPL but accumulated a set of non-acetylated GPL-3 isomers substituted by two, three or five methoxy groups (Fig. 4*B*).

### *M. abscessus* lacking *atf1*, *atf2*, and *MAB_1725c* synthesizes mono-*O*-acetylated GPL-3

Next, we examined in detail the GPL pattern of the triple Δ1,2,1725c mutant that showed a more complex pattern than the quadruple Δ1,2,1725c,3448 mutant, presumably because of the presence of partially acetylated products. The polar lipids from Δ1,2,1725c were separated by preparative TLC into four fractions, designated Δ1,2,1725c-F1 to Δ1,2,1725c-F4 (Fig. 4*C*), and subjected to MALDI-MS and MS/MS analysis (Fig. 4*D*). Fraction Δ1,2,1725c-F1 was identified as non-acetylated GPL-3 with a terminal Rha residue based on identical Rf, MALDI-MS, and MALDI-MS^2^ fragmentation patterns than Δ1,2,1725c,3448-F1 (Figs. 4, *C* and *D*; Fig. S9, *A* and *B*). Similarly, based on TLC mobility, MS, MS^2^, and MS^3^ analyses (Figs. 4, *C* and *D* and S8*C*), Δ1,2,1725c-F4 was identified as non-acetylated GPL-3 Ala-C_28_ with a terminal tri-*O*-methyl Rha, as observed in Δ1,2,1725c,3448-F3. In contrast, Δ1,2,1725c-F2 and Δ1,2,1725c-F3 were shown to contain differently methylated mono-*O*-acetylated GPL-3 species. In particular, Δ1,2,1725c-F2 was characterized by a set of three ions dominated by a signal at *m/z* 1362 (Fig. 4*D*). MS^2^ analysis of parent ion at *m/z* 1362 showed the presence of a complex mixture consisting of two major GPL-3 isomers with valinol- or alaninol-containing peptides identified as mono-*O*-acetylated GPL-3 Val-C_26_ and GPL-3 Ala-C_28_ substituted by terminal Rha residues (Fig. S10*A*). Alaninol- and valinol-containing peptides were differentiated by simultaneous detection of fragment ions at *m/z* 489 and 517 and those resulting from the cleavage of terminal glycosidic linkages at *m/z* 1156 and 1216. The second major ion in Δ1,2,1725c-F2 observed at *m/z* 1390 was identified as mono-*O*-acetylated GPL-3 Val-C_28_ according to primary fragment M-206 at *m/z* 1184 while the fragment M-146 at *m/z* 1244 confirmed the presence of a terminal Rha residue (Fig. S10*B*). Fraction Δ1,2,1725c-F3, that is exclusively observed in Δ1,2,1725c showed three major ions at *m/z* 1348, 1376, and 1404 by MALDI-MS (Fig. 4*D*) that were further subjected to MS^2^ and MS^3^ experiments. Of interest, the MS^2^ spectrum of parent ion at *m/z* 1376 yielded signals at *m/z* 1170 and 1216 generated by the cleavage of terminal mono-*O*-acetylated 6-dTal and mono-*O*-methyl Rha residues (Fig. S11*A*), as confirmed by MS^3^ fragmentation of ion at *m/z* 1170. MS^3^ Y2-fragment ions at *m/z* 503 or 531 and secondary fragments produced by Y3-fragments at *m/z* 792 or 820 typified two GPL-3 isomers Val-C_26_ and Ala-C_28_, both characterized by the presence of mono-*O*-acetylated 6-dTal and terminal mono-*O*-methyl Rha. The 28 a.m.u higher mass ion at *m/z* 1404 was similarly identified as GPL-3 Val-C_28_ based on MS^2^ analysis (Fig. S11*B*). The exclusive presence of *O*-acetyl groups in fractions Δ1,2,1725c-F2 and Δ1,2,1725c-F3 was further confirmed by ^1^H NMR analyses that showed a clear signal attributable to CH_3_-CO- group at δ2.14 ppm (Fig. S12). However, detailed NMR analysis of mono-acetylated GPL-3 did not reveal the exact position of the acetyl group due to the low relative amount of purified compound and possible heterogeneity caused by concomitant acetylation and methylation (data not shown). Overall, detailed GPL mapping in the multiple *atf*-deleted strains established that the loss of at least three *atf* genes presents inside and outside the *gpl* locus is required to significantly reduce GPL acetylation while disruption of all four *atf* genes is needed to abrogate GPL acetylation in *M. abscessus*.

### Deletion of all four *atf* genes disturbs the synthesis of GPL glycan moiety

**Figure 5:**
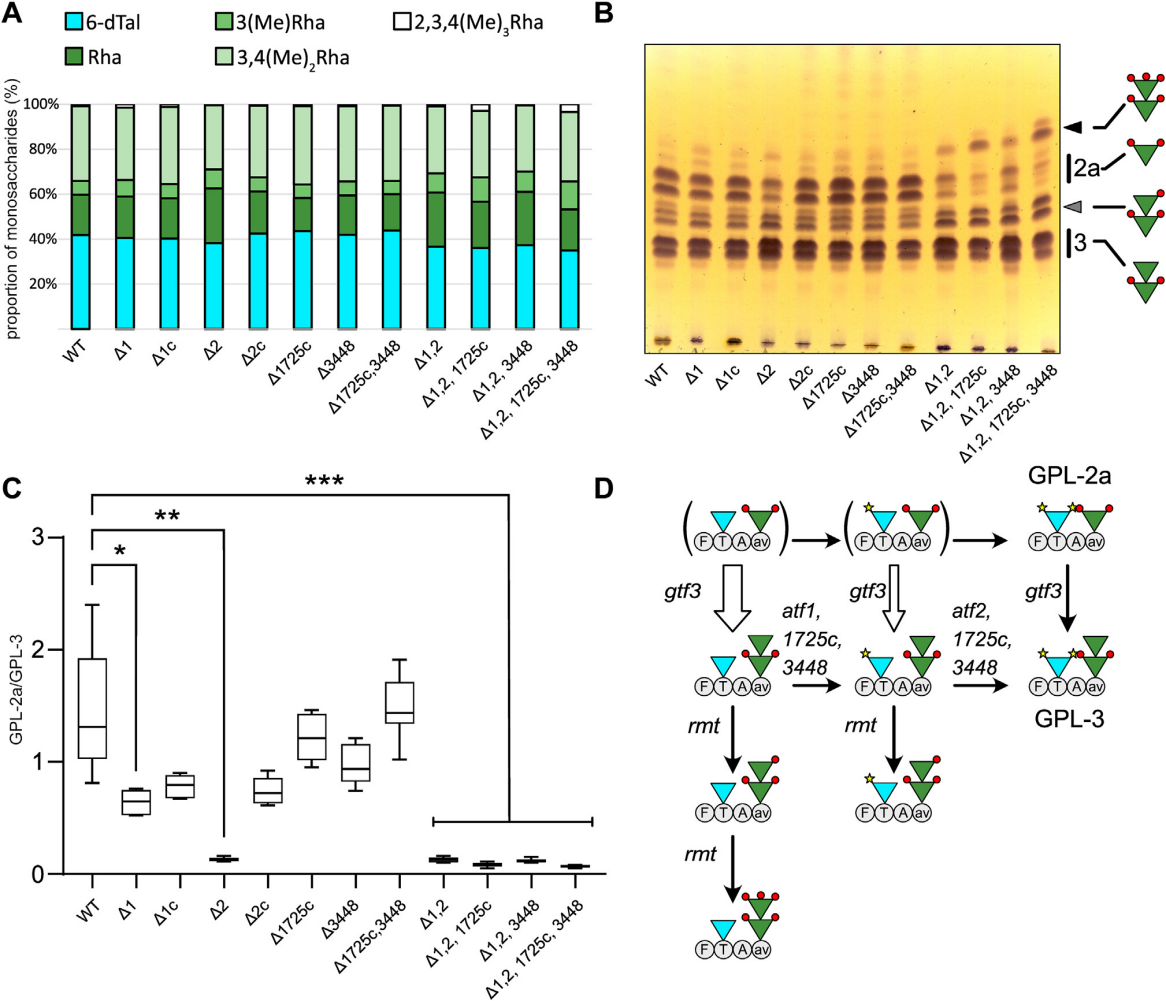
**Deletion of the *atf* genes alters the GPL pattern.***A*, monosaccharide composition of purified deacetylated GPL. The relative proportion of Rha residues increased in all mutants lacking *atf2*, as well as *O*-methyl Rha residues in Δ1,2,1725c and Δ1,2,1725c,3448. *B*, TLC of the saponified polar lipid fraction highlights important GPL modifications in all mutants deleted in *atf1* and *atf2* genes. Deacetylated dGPL-2a and dGPL-3 were attributed owing to saponification of the purified GPL-2a and GPL-3 as well as purified fractions from Δ1,2,1725c and Δ1,2,1725c,3448 (Fig. S13, *A* and *B*). The composition of rhamnan moiety is shown to the *right* of the TLC. *C*, dGPL-2a/dGPL-3 ratios in all mutant strains were established by integrating relative intensities of ions at *m/z* 1174 *versus* 1320 as well as 1202 *versus* 1348. In all mutants lacking *atf2* gene, the relative proportion of GPL-2a is significantly reduced. Median values are shown and results (n = 8) were analyzed using the Friedman *t* test. ∗*p* ≤ 0.05, ∗∗ *p* ≤ 0.01, ∗∗∗*p* ≤ 0.001. *D*, proposed biosynthetic pathway of GPL modification by glycosylation (*gtf*), acetylation (*atf*), and methylation (*rmt*). Acetyl groups are illustrated by *yellow stars* on the 6-dTal and *O*-methyl groups by *red circles*. All four identified acetyltransferases show partial overlapping activities. Atf1 produces a mono-*O*-acetylated GPL-3 while Atf2 catalyzes the transfer of the second acetyl group. MAB_1725c and MAB_3448 are likely to be active on both non-acetylated and mono-*O*-acetylated GPL, although with different efficiencies. The rhamnosyltransferase Gtf3 is thought to be more active on non-acetylated substrates (indiciated by a *thick arrow*), which would explain the accumulation of under-acetylated GPL-3, which in turn, serves as a substrate for rhamnosyl-methyltansferases (Rmt) to synthesize the over-methylated GPL-3 species. Under-acetylated GPL-2a (in *brackets*) are not observed in any of the studied strains as they represent biosynthetic intermediates that do not accumulate.

To address whether acetylation or acetyltransferases expression regulates the synthesis of GPL glycan moiety, the relative quantity of each deoxyhexose was determined in the various *atf* mutants. To do so, GPL was first chemically deacetylated to afford a complete purification from acyl ester glycolipids such as trehalose-based compounds, in particular DAT. Then, the monosaccharide composition of the resulting purified deacetylated GPL (dGPL) was established by GC/MS (Fig. 5*A*). This revealed that the overall proportion of Rha residues slightly increased in all mutants lacking *atf2* (below 60% in WT and up to 65% in Δ1,2,1725c,3448) but remained unchanged in all other mutants as well as in the Δ2-complemented strain. In agreement with the above-mentioned data, GC/MS analysis revealed the presence of about 3% 2,3,4 tri-*O*-methyl Rha in Δ1,2,1725c and Δ1,2,1725c,3448, but not in the other strains.

As saponification releases the acetyl groups, the migration of the resulting dGPL on TLC exclusively relies on the rhamnan moiety (Fig. 5*B*) which considerably simplifies the migration pattern as compared to their corresponding native GPL (Fig. 2*A*). Standard dGPL-3 and dGPL-2a were obtained by saponification of GPL-3 and GPL-2a (Fig. S13*A*) while standard deacetylated tri- and penta-*O*-methylated-GPL-3 were obtained from Δ1,2,1725c,3448-F2 and F3 (Fig. S13*B*). Based on the mobility of those standard molecules, the TLC analysis of deacetylated GPL fractions confirmed that the relative quantity of dGPL-2a was strongly decreased in all *atf2-*deficient mutants, with a complete loss of dGPL-2a in the quadruple mutant (Fig. 5*B*). Simultaneously, di-, tri-, and penta-*O*-methylated dGPL-3 strongly accumulated. The impact of *atf* deletion on reduced dGPL-2a production was quantified by computing the integration values of MALDI-MS diagnosis ion signals at *m/z* 1320 and 1348 for dGPL-3 and 1202 and 1174 for dGPL-2a (Fig. S13*C*). Determination of the dGPL-2a/dGPL-3 ratio clearly indicated that the deletion of *atf2* was sufficient to reduce, by at least 10-fold, the relative quantity of GPL-2a with respect to GPL-3 (Fig. 5*C*). In comparison, the individual deletion of *atf1*, *MAB_1725c*, or *MAB_3448* failed to or only marginally reduced the GPL-2a content.

To investigate the specificity of the individual acetyltransferases in *M. abscessus,* each gene was reintroduced into the Δ1,2,1725c,3448 quadruple mutant and the corresponding GPL profiles were analyzed. Western blotting using anti-HA antibodies clearly indicated that all four proteins were produced in the Δ1,2,1725c,3448 mutant, albeit at different levels (Fig. S2*C*). As shown by TLC and MS analyses, expression of the individual genes, with the exception of *atf2*, significantly altered the GPL profiles as compared to the Δ1,2,1725c,3448 quadruple mutants, but did not modify the apolar lipid pattern (Fig. S14, *A* and *B*). TLC and MS analysis of the deacetylated GPL fractions of the four mutant strains were dominated by dGPL-3 as in the parental Δ1,2,1725c,3448 mutant strain, confirming that the expression of individual genes only impacts on acetylation (Figs. S13*C* and S14*C*). Moreover, the expression of the single acetyltransferases did not rescue the WT expression level of GPL-2a. Analysis of the native GPLs of the Atf1-expressing strain showed that it accumulates mono*-O-*acetylated GPL-3, demonstrating that Atf1 can transfer a single acetyl group onto unacetylated GPL3, but appears to be unable to use the produced mono-*O*-acetylated GPL-3 as a substrate (Fig. S14, *B* and *C*). In contrast, the GPL profile of the strain expressing Atf2-shows predominantly deacetylated and hypermethylated GPL3 as in the quadruple mutant, suggesting that Atf2 cannot use a non-acetylated substrate (Fig. S14, *B* and *C*). The unique expression of *MAB_1725c* or *MAB_3448* correlates with the disappearance of non-acetylated GPL3 and the concomitant synthesis of mono- and di-*O*-acetylated GPL3. This demonstrates that both gene products are capable of catalyzing the acetylation of non-acetylated or mono*-O-*acetylated substrates, although with different efficiencies, with the strain expressing *MAB_1725c* showing greater completeness for GPL acetylation.

### Disruption of acetyltransferases influences colony morphology but not the uptake of *M. abscessus* by macrophages

**Figure 6:**
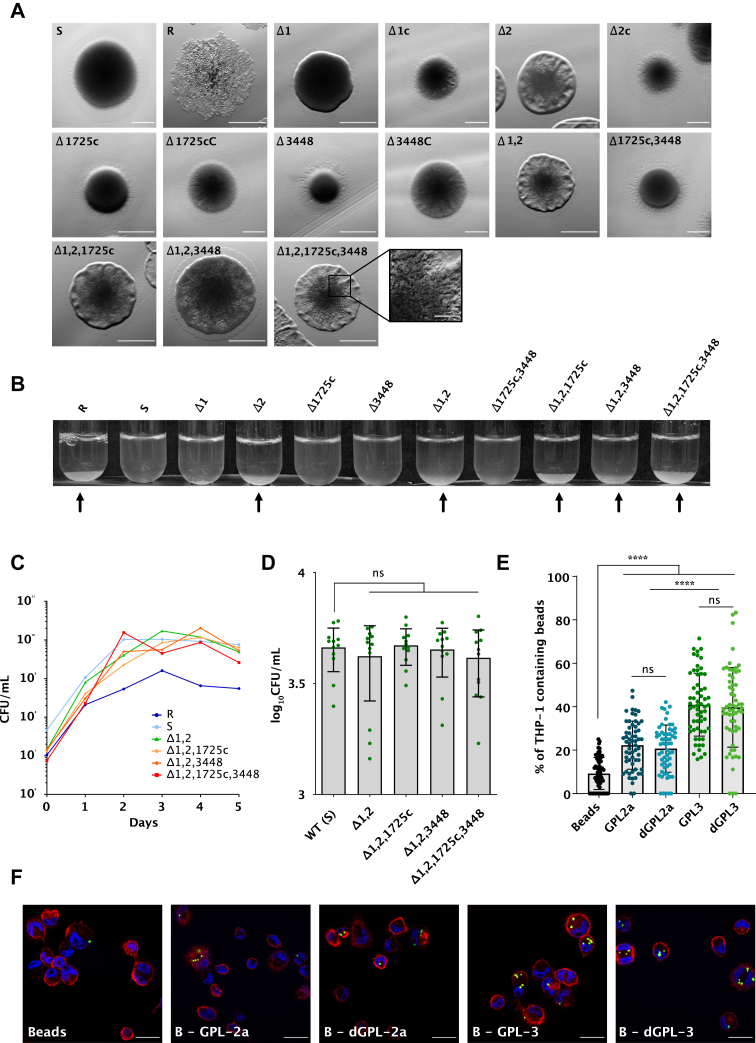
**Impact of GPL acetylation on bacterial growth, morphology and sedimentation.***A*, colony morphology of the wild-type *M. abscessus* smooth and rough strains, *atf* mutants, and complemented strains. Scale bars represent 1 mm. Scale bar of inset represents 0.2 mm. *B*, sedimented bacterial aggregates are indicated by *arrows*. *C*, growth curve of reference strains (S and R), Δ1,2, Δ1,2,1725c, Δ1,2,3448, and Δ1,2,1725c,3448 at 37 °C for 5 days with agitation. The experiment was performed three times. *D*, internalization by macrophages of different *atf* mutants and wild-type strain (S) after 3 h of infection (MOI 2:1). Intracellular bacteria are expressed as CFU/ml and error bars represent standard deviations. Four independent experiments performed in triplicate are plotted on the graph and analyzed using the two-tailed Mann–Whitney *t* test. *E*, the influence of GPL acetylation in macrophage infection was evaluated with fluorescent beads coated with di- (GPL-2a) or tri-glycosylated (GPL-3) GPL, either di- or deacetylated (dGPL) using ten beads/cell. Non-coated beads were included as a control group. Each symbol represents the percentage of bead-containing macrophages in one field. Experiments were conducted three times (n = 60) and data are expressed as mean values ± SD. Results were analyzed using the Mann-Whitney *t* test. ns, non-significant; ∗∗∗∗*p* ≤ 0.0001. *F*, representative fields of fluorescent beads (*green*) internalized by THP-1 cells. DAPI (*blue*) was used to stain the nuclei and CD43 antibodies (*red*) to detect the cell membrane. Scale bar, 50 μm.

It is well established that the absence or the presence of GPL determines the morphotype of *M. abscessus* colonies on agar medium (5, 16, 33). In addition, that a *gtf1* mutant lacking 6-dTal acquires a rough morphotype (15), prompted us to compare the colony morphology of the single, double, triple, and quadruple *atf* mutants. Observation of individual colonies on Tryptic Soy agar failed to reveal morphological differences in Δ1, Δ1725c, Δ3448 and Δ1725c,3448 and all appeared similar to the parental S strain (Fig. 6*A*); none of these have altered acetylation patterns. Unexpectedly, Δ2 and Δ1,2 showed a different morphology than the parental S strain but distinct from the typical rough and dry features of the R strain. Strains Δ1,2,1725c, Δ1,23,448, and Δ1,2,1725c,3448 all exhibited morphological differences, possibly as a consequence of the reduced GPL-2a/GPL-3 ratio (Fig. 6*A*). In contrast to the Δ*gtf1* mutant lacking 6-dTal with a pronounced corded phenotype (15), none of the acetylation mutants displayed the typical serpentine cords of the R variant (data not shown), suggesting that the corded structure of Δ*gtf1* is primarily caused by the loss of the 6-dTal rather than by the absence of the acetyl substituents. Similar to the *M. abscessus* R strain, only mutants lacking *atf2* rapidly sedimented in liquid culture, again underscoring a possible role of the reduced GPL-2a/GPL-3 ratio in bacterial aggregation (Fig. 6*B*). The *in vitro* growth curves of the *atf* mutants in 7H9 broth at 37 °C were comparable to the one of the parental S strain, reaching a plateau after 2 days (Fig. 6*C*). This suggests that the single or multiple deletions of the *atf* genes do not influence the replication rate of *M. abscessus* in planktonic cultures.

We next infected human THP-1 macrophages for 3 h with *M. abscessus* S, Δ1,2, Δ1,2,1725c, Δ1,2,*3*448, and Δ1,2,1725c,3448 prior to assessing phagocytosis by CFU counting. As shown in Figure 6*D*, all strains were equally taken up by macrophages, suggesting that GPL acetylation is not required for the internalization of *M. abscessus* by macrophages. To confirm these observations, we determined the contribution of GPL acetylation in phagocytosis using acetylated *versus* deacetylated GPL-2a and acetylated *versus* deacetylated GPL-3-coated fluorescent beads. Quantification of the proportion of bead-containing cells indicated that macrophages internalized significantly more GPL-2a-coated beads than uncoated beads (Fig. 6*E*). As reported previously, THP-1 cells phagocytosed more GPL-3-coated beads than GPL-2a-coated beads, thus highlighting the contribution of the terminal Rha in this process (15). However, no significant differences between dGPL-2a *versus* GPL-2a or between dGPL-3 *versus* GPL-3 were noticed (Fig. 6*E*). High-resolution confocal imaging confirmed the intracellular localization of the different GPL-counted beads (Fig. 6*F*).

Overall, the structure-function comparison using either whole bacilli or purified GPL beads suggests that acetylation of 6-dTal is not essential for the uptake of *M. abscessus* by macrophages.

## Discussion

Acetylation of GPL has been proposed to regulate various biological processes such as sliding motility and biofilm formation in *M. smegmatis* (36) or macrophage apoptosis in *M. abscessus* through the interaction of di-*O*-acetylated GPL with mitochondrial cyclophilin D (13). While *M. smegmatis* produces essentially di-glycosylated GPL-2, *M. abscessus* synthesizes equivalent amounts of GPL-2a and GPL-3, which can be easily distinguished by TLC and mass spectrometry. Previous work demonstrated that disruption of the unique *atf* gene in *M. smegmatis* abolished di-*O*-acetylated GPL-2 synthesis (8, 36). However, in the present work, our attempts to quantitatively inhibit GPL acetylation in *M. abscessus* by simultaneously deleting *atf1* and *atf2*, located within the *gpl* biosynthetic locus, were unsuccessful despite the fact that Atf1 and Atf2 sequentially transfer acetyl groups to 6-dTal when overexpressed in the *M. smegmatis atf* mutant (8). Indeed, single and simultaneous disruptions of *atf1* and *atf2* genes only resulted in a slight decrease of GPL acetylation. These observations challenge the initial hypothesis that *atf1* and *atf2* are sufficient to sequentially add the two acetyl groups on the 6-dTal in *M. abscessus*. Unexpectedly, disruption of *atf2* induced a notable decrease of di-*O*-acetylated GPL-2a correlating with an accumulation of mono- and di-*O*-acetylated GPL-3. Since all mutants lacking *atf2* sedimented efficiently in liquid culture, similarly to *M. abscessus* R, it may be proposed that this aggregation property is linked to the increased proportion of GPL-3 over GPL-2a. This hypothesis is strengthened by the fact that *M. abscessus* S overproducing the rhamnosyl-transferase Gtf3, also associated with an increased GPL-3/GPL-2a ratio, aggregated rapidly in liquid culture like the original R strain (15).

We posit here the existence of additional acetyltransferases, encoded outside the *gpl* locus, capable of functionally substituting Atf1 and Atf2. To advance our understanding of the GPL acetylation genetic requirements, two additional Atf-related enzymes potentially involved in GPL *O*-acetylation were identified, *MAB_1725c* and *MAB_3448*. Whereas individual or simultaneous deletion of these genes did not significantly modify the GPL profile, multiple deletions of *atf1*, *atf2*, and *MAB_1725c* resulted in a high accumulation of mono-*O*-acetylated GPL-3. Further deletion of *MAB_3448* in Δ1,2,1725c,3448 induced complete loss of GPL acetylation, as assessed by the accumulation of de-*O*-acetylated GPL-3. In addition to the loss of 6-dTal acetylation in Δ1,2,1725c and Δ1,2,1725c,3448 strains, additional methylation of the terminal Rha residue occurred in GPL-3, generating several highly methylated by-products that are not observed in WT *M. abscessus*. Overall, observation of the individual GPL profiles of single and multiple mutants of *M. abscessus* reflects a complex compensatory mechanism between the different *atf* genes that does not exist in *M. smegmatis*. Accordingly, a bioinformatics analysis of the genomes of GPL-producing mycobacteria shows that while *M. smegmatis* and *Mycobacterium avium* have only one *atf*, *Mycobacterium chelonae* possesses two (*atf1* and *atf2*) (Table S4). Thus, the requirement for additional acetyltransferases in *M. abscessus* CIP104536^T^ appears to be a unique feature among the few mycobacterial species analyzed.

Three hypotheses for the biosynthesis of GPL in *M. abscessus* can be drawn from these observations, as summarized in Figure 5*D*. (**1**) The four putative acetyltransferases identified show a high level of redundancy in terms of activity toward GPL-3 and probably GPL-2a. Specifically, single and simultaneous deletions of *atf1* and *atf2* generate a modest accumulation of mono-*O*-acetylated GPL-3 while still producing di-*O*-acetylated GPL-3. Interestingly, when *atf1* or *atf2* are expressed individually in the quadruple mutant, this results in the production of mono-*O*-acetylated or non-acetylated GPL-3 rather than di-*O*-acetylated GPL-3 as in the WT strain. This strongly suggests that Atf1 and Atf2 are specific for non-acetylated and mono-*O*-acetylated GPL-3, respectively. In comparison, both MAB_1725c and MAB_3448 can acetylate non- and mono-*O*-acetylated GPL-3, although with different efficiencies, with MAB_1725c being the most active enzyme, as revealed by the comparison of the level of di-*O*-acetylated GPL-3 in the Δ1,2,1725c with the Δ1,2,3448 and in mutants that individually express MAB_1725c or MAB_3448. (**2**) Gtf3 has higher activity on non-acetylated GPL-2a than acetylated GPL. We have previously demonstrated that Gtf3 uses di-*O*-acetylated GPL-2a to synthesize GPL-3 by transferring an additional Rha onto the internal Rha residue (15). The observation that GPL-2a content was strongly reduced when the *atf* genes were deleted put into light a totally unexpected regulatory process occurring during GPL synthesis that cannot be directly explained by the intrinsic activities of the enzymes. However, it may be partially explained by a modification in the metabolic processing of GPL-2a due to increased activity of Gtf3 on non-acetylated GPL that would reroute the pool of GPL-2a toward GPL-3. If so, acetylation of GPL appears as a possible regulatory signal that maintains the GPL-2a/GPL-3 balance. It can also be hypothesized that the physical presence of acetyltransferases may limit the access of Gtf3 to GPL-2a and, consequently, restrict the production of GPL-3 due to steric hindrance. (**3**) In a similar manner, the build-up of under-acetylated GPL-3 in mutant strains is coupled with the unexpected accumulation of hyper-methylated species that are not found in the WT *M. abscessus* strain. It may be inferred that methylation acts as a compensatory mechanism to keep the overall hydrophobicity of GPL under the control of methyltransferases showing residual activity on under-acetylated GPL-3. Indeed, three genes *rmt2*, *rmt3*, and *rmt4* located within the *gpl* locus (Fig. 1) have orthologs in *M. smegmatis* and *M. avium* that are involved in the methylation of the first rhamnose (43, 44). Although attractive, these hypotheses should ideally be confirmed by studying the enzymatic activities of recombinantly produced acetyltransferases using individual acceptor substrates and identifying methyltransferases involved in the modification of GPL in *M. abscessus*.

Previous findings indicated that 6-dTal is a critical determinant promoting bacterial cell entry into macrophages (15), prompting us to investigate whether this was due to the loss of the monosaccharide and/or caused by the absence of the two acetyl groups. Quantitative analysis of the proportion of macrophages containing the various *atf* mutants at 3 h post-infection revealed that all strains were equally taken up by THP-1 cells, thus ruling out a role of GPL acetylation in the early interaction between the bacilli and the macrophage surface. This was also supported when determining the proportion of macrophages containing beads coated with chemically deacetylated GPL-2a *versus* di-*O*-acetylated GPL-2a or deacetylated GPL-3 *versus* di-*O*-acetylated GPL-3. Thus, the results obtained from whole bacteria or beads coated with purified GPL suggest that acetylation of GPL is not essential in the recognition between GPL and the macrophage receptor(s). Therefore, it can be speculated that the reduced uptake of the *gtf1* mutant is likely a consequence of the absence of the 6-dTal rather than of the acetyl groups. Given the importance of GPL-3 in the internalization of *M. abscessus* S by macrophages (15), one would expect the strains displaying high proportion of GPL-3 as compared to GPL-2a to be more efficiently internalized by THP-1 cells than the WT strain. However, this phenomenon was not observed in the case of the *atf2*-deletion mutants. This could be due to hyper-*O*-methylation of GPL compensating for the loss of acetylation during interaction between the bacilli and the host receptor(s). This hypothesis will be explored in future studies on the roles of the rhamnosyl-*O-*methyl transferases Rmt2, Rmt3, and Rmt4 in GPL structure and function.

An intriguing observation is that MAB_1725c is encoded by prophage prophiATCC19977-1, grouped within Subcluster MabA1. Prophages are prevalent and highly diverse among *M. abscessus* strains (41). However, *atf*-like genes are generally only present within Cluster MabA prophages, and only about half of all MabA prophages have an *atf* gene (41). These include prophage prophiGD17-2 and RNAseq analysis shows that its *atf* gene is lysogenically expressed – albeit at low levels – and can at least partially *O*-acetylate GPL in Δ1,2 (Fig. S15). Thus, at least in Δ1,2, the Atf encoded by the prophage participates in GPL biosynthesis and, therefore, in host cell wall assembly. These observations also suggest that GPL composition could vary among *M. abscessus* clinical isolates depending on the prophage status. Finally, we note that no *M. smegmatis* phages code for *atf* genes, but *Gordonia* phage Doggs and *Arthrobacter* phage Faja both code for Atf-like enzymes, and although GPL are not well characterized in these strains, it suggests that prophages may play broad roles in cell wall chemistry.

## Experimental procedures

### Mycobacterial strains, growth conditions, and reagents

All bacterial strains used for this study are listed in Table S3. Bacteria were grown in Middlebrook 7H9 broth (BD Difco) supplemented with 0.025% Tyloxapol and 10% oleic acid, albumin, dextrose, catalase (OADC enrichment) or on Middlebrook 7H10 agar (Difco) containing 10% OADC enrichment (7H10^OADC^) at 37 °C, with antibiotics, if required. A Bio-Rad Gene pulser (25 μF, 2500 V, 800 Ω) was used to transform electrocompetent mycobacteria. After transformation, strains carrying pTEC27 (Addgene, plasmid 30,182) were selected in the presence of 1 mg/ml hygromycin for pTEC27 plasmid and strains carrying the pMV306 derivatives were selected on 250 μg/ml kanamycin.

### Deletion of *atf* genes and complementation in *M. abscessus*

All deletion mutants were generated in the smooth (S) variant of the reference strain CIP104536^T^ using the suicide vector pUX1-*katG* by double homologous recombination (37). Briefly, the upstream and downstream gene regions were PCR-amplified using the primers listed in Table S2 and ligated into the PacI/NheI-linearized pUX1-*katG*. After transformation, bacteria were selected on 7H10^OADC^ supplemented with 250 μg/ml kanamycin with a visual screening of red fluorescent colonies, which have undergone the first homologous recombination. The second homologous recombination event was induced by INH counter-selection and selected on 7H10^OADC^ with 50 μg/ml INH and screening for non-fluorescent colonies (15, 37). This unmarked deletion system allowed to successively delete all four *atf* genes in the *M. abscessus*. Complementations of the single and quadruple *atf* mutants were done by introducing the complementation plasmids generated using the integrative pMV306 (Table S1). Genes in fusion with an HA-tagging sequence were amplified by PCR under the control of the *hsp60* promoter (15). Proper gene deletion and all plasmids were verified by DNA sequencing.

### Western blotting

Bacteria were harvested, resuspended in PBS and protease inhibitor, and disrupted by bead beating using 1 mm diameter glass beads and a Mixer Mill MM 301 (Retsch, Germany) for two pulses of 3 min at 30 Hz. Protein concentration was assessed using the BCA Protein Assay Reagent kit (Pierce), according to the manufacturer’s instructions. Equal amounts of proteins (10 μg) were separated by 12% SDS-PAGE, transferred onto a nitrocellulose membrane, probed for 1 h with either rat anti-HA (dilution 1:1000; Sigma) or rat anti-KasA (dilution 1:2000; loading control) antibodies. Membranes were washed and incubated for 45 min with goat anti-rat antibody conjugated to HRP (dilution 1:5000; Abcam). Bands were revealed using a SuperSignal West Femto (ThermoFisher Scientific) and a ChemiDoc MP system (Bio-Rad laboratories).

### Colony morphology, sedimentation, and growth

Colony morphology was assessed from log phase cultures (OD_600_ = 1) and streaked on Tryptic Soy agar. Plates were incubated 4 days at 37 °C and then imaged using a Zeiss microscope equipped with a Zeiss Plan Neo Fluor Z13/0.25 FWD objective. Images were taken with an Axiocam503 monochrome (Zeiss) camera and processed using ZEN 2 (blue edition). Sedimentation of bacterial aggregates was followed as reported earlier (24). Growth was initiated by inoculating mid-log phase cultures into fresh 7H9^OADC^ at an OD_600_ of 0.05. Cultures were incubated at 37 °C with shaking and a sample of 1 ml of serially diluted culture was harvested every day and plated onto LB agar. After 4 days at 37 °C, colonies were counted to determine the colony forming unit (CFU).

### Glycolipids extraction

Bacteria grown on 7H10^OADC^ agar plates without detergent were collected and lyophilized. 50 mg of bacterial pellets were weighted and apolar lipid fractions were first extracted in order to avoid TDM interference. GPL were extracted from the polar lipid fraction, first with chloroform/methanol/0.3% NaCl (9/10/3, v/v/v) and then with chloroform/methanol/0.3% NaCl (5:10:4, v/v/v). The combined solvent extracts were mixed for 5 min with chloroform and 0.3% NaCl (1:1, v/v) and centrifuged at 3000*g* for 5 min to separate the lower organic phase from the aqueous phase. The upper aqueous layer was discarded and the lower organic phase was evaporated under a stream of nitrogen and resuspended in chloroform/methanol (2:1, v/v).

### TLC analysis

Apolar and polar lipids were subjected to TLC analysis. 10 μl of extract were spotted along 0.5 mm lane with glass capillary on Silica gel 60 F_254_ plates (Merck). GPL were separated in one or two migrations at 4 °C using chloroform/methanol/water (90:10:1, v/v/v) and more polar lipids were visualized with chloroform/methanol/water (65:25:4 or 30:8:1, v/v/v). Glycolipids were revealed by spraying the plates with orcinol in 20% sulfuric acid and charring.

### GPL purification

Preparative TLC was performed on polar extracts: 300 μl were spotted on a 160 mm lane on a 60 μm silica gel plate with a glass back (20 cm × 20 cm) and migrated in a solution of chloroform/methanol/water (90:10:1, v/v/v). Plates were reversibly stained with iodide vapor to label bands, which were scrapped and GPL was further extracted in chloroform/methanol (2:1, v/v) under sonication for 1 h. Silica was, respectively, filtrated on glass wool and through a 0.2-μm PTFE syringe filter.

### Glycolipids saponification

In all, 300 μl of the polar lipid fraction were dried under nitrogen, 1 ml sodium hydroxide 0.1 M in chloroform/methanol (1:1, v/v) was added, and heated overnight at 37 °C. 1 ml butanol and 1 ml water were added and the mixture vortexed for 1 min and centrifuged for 30 s. The upper butanolic phase was dried under nitrogen and dissolved in 300 μl chloroform/methanol (2:1, v/v).

### Itol-acetates derivation

For the hydrolysis step, 1 μg mesoinositol was added to the deacetylated GPL fraction then, 1 ml 3 M TFA was mixed, heated 4 h at 80 °C, dried and desiccated overnight. The reduction step was conducted for 4 h at room temperature in 500 μl NaBH_4_ 10 mg/ml in 2 M NH_4_. The reaction was stopped with concentrated glacial acetic acid. Samples were dried at 55 °C under a nitrogen stream by co-distillation with methanol/acetic acid five times, dessicated overnight. Peracetylation was done by incubation in 500 μl anhydride acetic 4 h at 80 °C. The reaction products were extracted several times with chloroform/water. The chloroform-rich phase was then filtered, dried and dissolved in 500 μl chloroform. For GC-FID, 1 μl of itol-acetate derivatives was injected in splitless mode with an automatic sampler on a Solgel 1 MS 30 m × 0.25 mm × 0.25 μm capillary column with the following gradient temperature: 120 to 230 °C at 3 °C/min, then to 270 °C at 10 °C/min. Compounds were detected with a flame ionization detector on a HP-7820 gas chromatograph (Agilent Technologies). Previously determined retention times were used to identify each deoxyhexoses (15).

### MALDI-TOF mass spectrometry

Before spotting 1 μl on the MALDI plate with a glass capillary tube, 10 μl of 20 mg/ml dihydroxybenzoïc acid (DHB) in chloroform/methanol (1:2, v/v) were mixed with 10 μl of the sample extract in chloroform/methanol (2:1, v/v). MS and MS^n^ spectra were acquired on an Axima Resonance (Shimadzu, Kyoto, Japan) in reflectron mode. For MS^2^ experiments, ion selection was set from 250 to 500 Δm, and collision energy was tuned from 300 eV to 600 eV. The GPL-2a/GPL-3 ratio was determined by dividing the relative intensities of the detected ion’s pair at *m/z* 1174 and 1320 or 1202 and 1348 following 1/2, 1/3, or 1/5 sample dilution with the matrix.

### Nuclear magnetic resonance

TLC-purified GPL was dried and dissolved in a mixture of CDCl_3_/CD_3_OD (2:1, v/v) with 0.03% trimethylsilane (Eurisotop, France) three times and then dissolved in a final volume of 270 μl. Samples were introduced into a 3 mm glass tube (Shigemi). A TBI probe was used to observe ^1^H and ^13^C nuclei at 293K on an AVANCE II system (Bruker Biospin GmbH). Impulsion sequences used for homonuclear and heteronuclear experiments were from the manufacturer. After acquisition, phase correction and calibration on methanol signals were performed for δ ^1^H and δ ^13^C.

### Internalization of *M. abscessus* by macrophages

THP-1 macrophages were grown in RPMI medium supplemented with 10% fetal bovine serum (Sigma-Aldrich) (RPMI^FBS^), differentiated in the presence of 20 ng/ml phorbol myristate acetate in 24-well flat-bottom tissue culture microplates (10^5^ cells/well) and incubated for 48 h at 37 °C with 5% CO_2_. Infections with *M. abscessus* strains were performed for 3 h at 37 °C in the presence of 5% CO_2_ with two bacteria per cell (MOI 2:1). Cells were carefully washed three times with PBS and then incubated with RPMI^FBS^ supplemented with 250 μg/ml amikacin for 2 h to kill extracellular bacteria. The medium containing amikacin was discarded and cells were washed three times with PBS prior to assess CFU by lysing cells with 100 μl of 1% Triton X100 and plating serial dilutions of the homogenates. CFU were counted after 4 days of incubation at 37 °C, as described earlier (15).

### Fluorescent beads phagocytosis assay

Fluorescent beads were coated with purified acylated and deacylated GPL-2a and GPL-3, as reported previously (15). THP-1 cells were incubated for 4 h at 37 °C with fluorescent beads (ten beads/macrophage), washed and stained using anti-CD43 antibodies and Alexa Fluor 594-coupled anti-mouse secondary antibody and DAPI. The percentage of macrophages containing beads was quantified using an epifluorescence microscope (15).

### Statistical analyses

Statistical analyses were carried out with Prism 9.0 (Graphpad). Details are given in the legend of each figure. ns, *p* ≥ 0.05, ∗*p* ≤ 0.05, ∗∗*p* ≤ 0.01, ∗∗∗*p* ≤ 0.001, ∗∗∗∗*p* ≤ 0.0001.

## Data availability

All data are contained within the manuscript and Supporting information section. The raw data can be shared upon request to yann.guerardel@univ-lille.fr.

## Supporting information

This article contains supporting information (15, 24, 25, 38, 41).

## Conflict of interest

The funders had no role in study design, data collection, interpretation, or the decision to submit the work for publication. G. H. receives support through a collaborative research agreement with Janssen Inc.
