# Supplementary material for: Multiple Mycobacterium abscessus O-acetyltransferases influence glycopeptidolipid structure and colony morphotype
Source: J Biol Chem. 2023 Jun 28;299(8):104979. doi: 10.1016/j.jbc.2023.104979 (PMC10400925; doi:10.1016/j.jbc.2023.104979)
Supplement: Supporting Figures S1–S15 and Tables S1–S4 [file mmc1.docx]

**Supporting information**

**Multiple *Mycobacterium abscessus* *O*-acetyltransferases influence**

**glycopeptidolipid structure and colony morphotype**

Morgane Illouz^1,&^, Louis-David Leclercq^2,&^, Clara Dessenne^2^, Graham Hatfull^3^, Wassim Daher^1,4^,

Laurent Kremer^1,4,#^, and Yann Guérardel^2,5#^

^1^Centre National de la Recherche Scientifique UMR 9004, Institut de Recherche en Infectiologie de Montpellier (IRIM), Université de Montpellier, 1919 route de Mende, 34293, Montpellier, France.

^2^Univ. Lille, CNRS, UMR 8576 - UGSF - Unité de Glycobiologie Structurale et Fonctionnelle, F-59000 Lille, France.

^3^Department of Biological Sciences, University of Pittsburgh, Pittsburgh, Pennsylvania, US

^4^INSERM, IRIM, 34293 Montpellier, France.

^5^Institute for Glyco-core Research (iGCORE), Gifu University, Gifu, Japan

^&^These authors contributed equally to this work.

**^#^**For correspondence: E-mails [yann.guerardel@univ-lille.fr](mailto:yann.guerardel@univ-lille.fr), Tel: (+33) 3 20 43 69 41; [laurent.kremer@irim.cnrs.fr](mailto:laurent.kremer@irim.cnrs.fr), Tel: (+33) 4 34 35 94 47.

**Running title:** Acetylation of GPL in *Mycobacterium abscessus*

**Keywords:** *Mycobacterium abscessus*, glycopeptidolipid, cell wall, acetyltransferase, macrophage, mass spectrometry.

**SUPPLEMENTARY FIGURES**


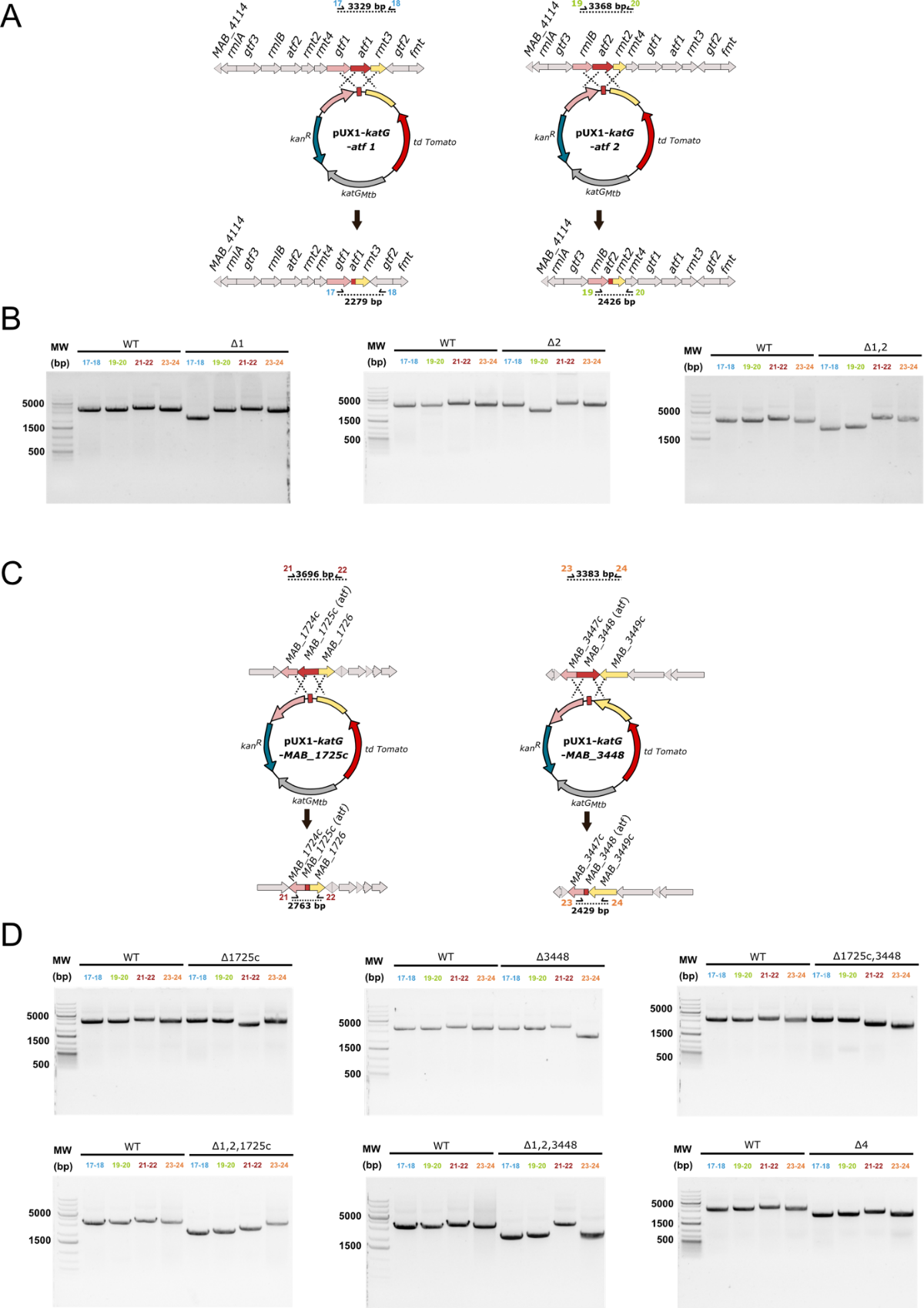


**Figure S1: Generation of the *atf* mutants in *M. abscessus*. (A, C)** The gene of interest to be inactivated in the bacterial chromosome is colored in red. The upstream and downstream regions of the gene of interest are in pink and yellow, respectively. Crosses represent homologous recombination events. Primers used are listed in **Table** **S2**. The intact locus is drawn above the plasmid and the modified locus is shown below the plasmid. **(B, D)** PCR profile of the different mutants compared to WT strain. To amplify the different loci, genomic DNA was used as template. Sequencing of the amplicons was performed to confirm the proper deletions.

**Figure S2: Acetyltransferase complementation in *M. abscessus* mutants. (A)** Diagrams of the genes of interest fused with an HA-tag on the C-terminal side produced under the control of the *hsp60* promoter. The molecular weight of the protein with the HA-tag is indicated below each diagram. **(B, C)** Western blot analysis showing the production of the four HA-tagged Atf proteins in the single **(B)** and quadruple **(C)** *atf* mutants (upper panels). The KasA protein serves as an internal loading control (lower panels).


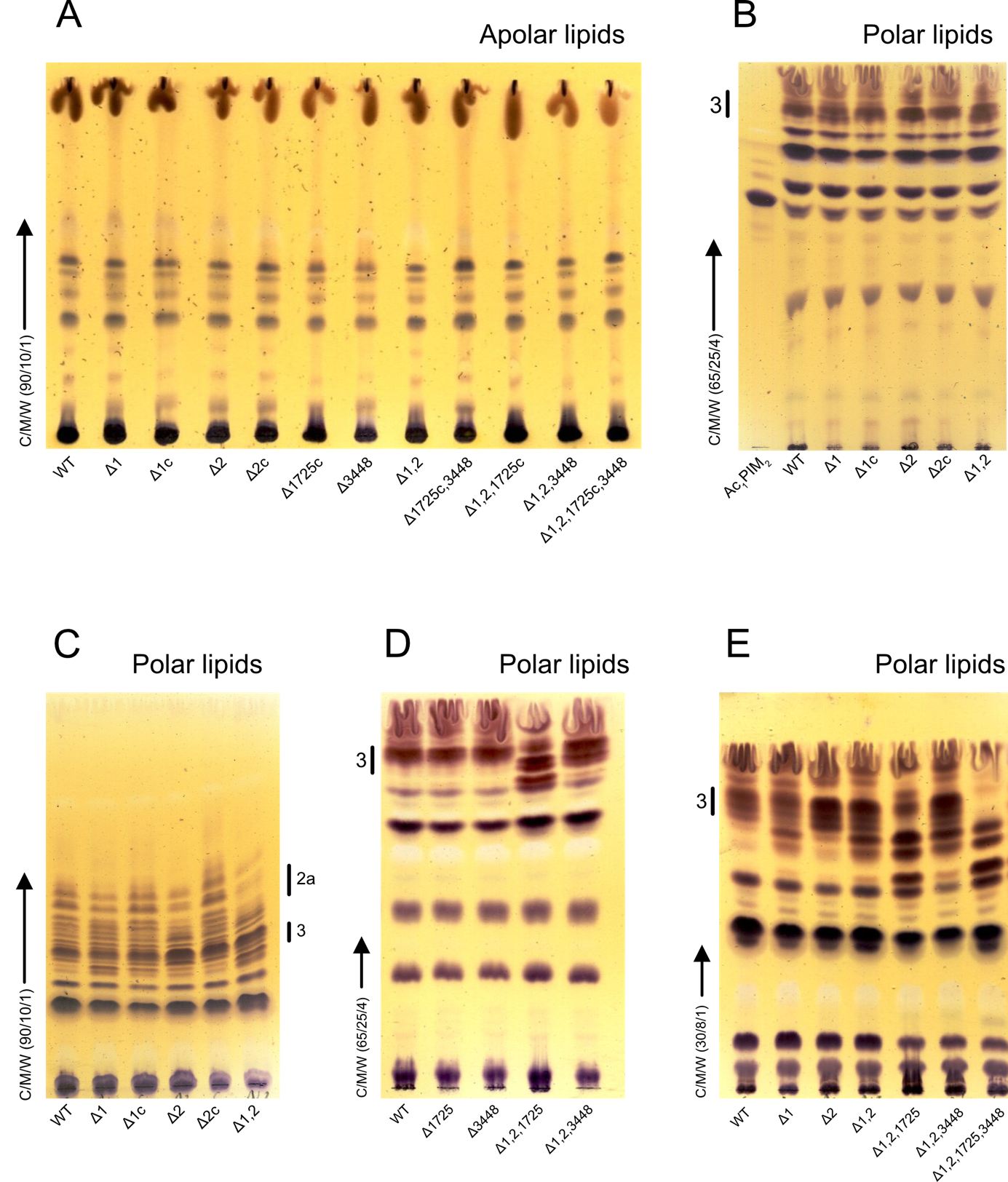


**Figure S3: TLC analysis of polar and apolar fractions from mutant strains developed in different solvents**. **(A)** Petroleum ether extracted apolar lipid fraction of wild-type (WT) and all acetyltransferase mutant strains. **(B, C)** Polar glycolipids from WT, Δ1 and complemented strain, Δ2 and complemented strain, Δ1,2 showed phosphatidyl *myo*-inositol mannosides and GPLs. **(D, E)** Polar glycolipids from WT, Δ1, Δ2, Δ1725c, Δ3448 as well as Δ1,2 and Δ1,2,1725c and Δ1,2,3448 mutants. Only GPL showed significant modifications in the mutant strains.


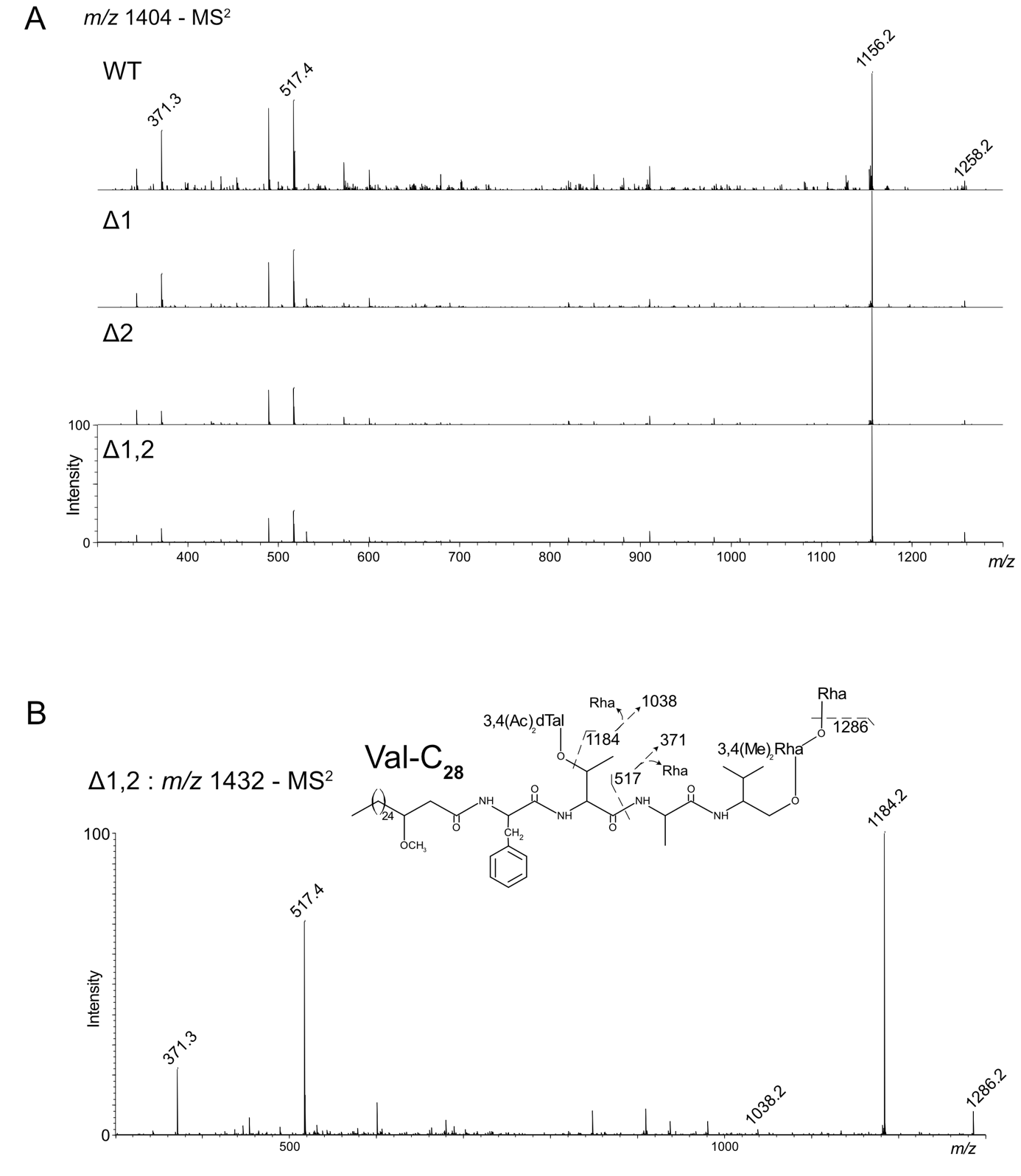


**Figure S4: (A)** MALDI-MS^2^ spectra in positive mode of the selected parent ion at *m/z* 1404 from WT, Δ1, Δ2 and Δ1,2 mutant strains. **(B)** MALDI-MS^2^ spectra in positive mode of the selected parent ion at *m/z* 1432 of Δ1,2. Fragmentation scheme shows the presence of 3,4 di-*O*-acetylated 6d-Tal in valinol form with C28 lipid (Val-C_28_).


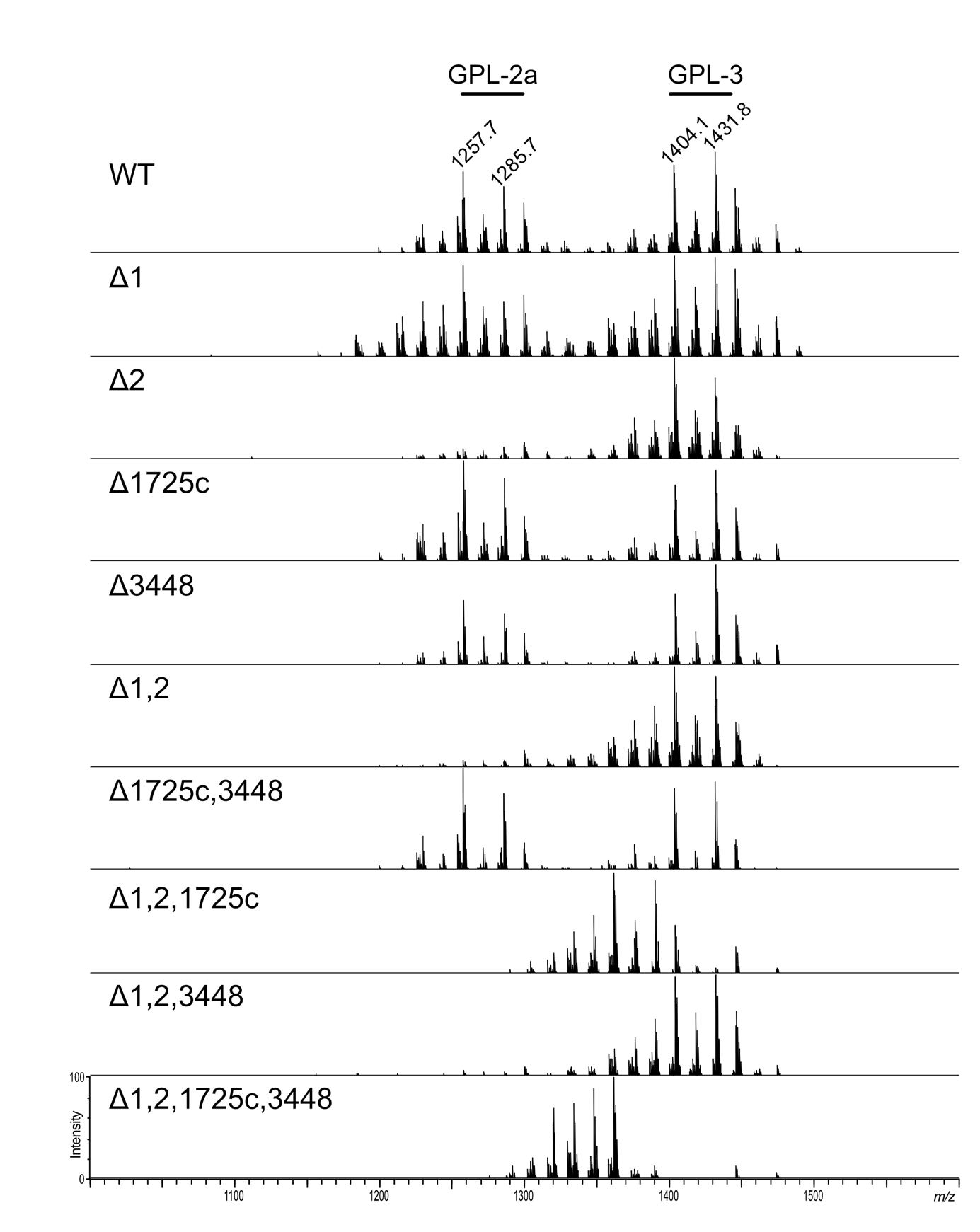


**Figure S5: MALDI-MS spectra in positive mode of the polar lipid fraction from wild-type and acetyltransferase mutants**. Previously characterized GPL-2a and GPL-3 (15, 24) are shown on top of the spectra.


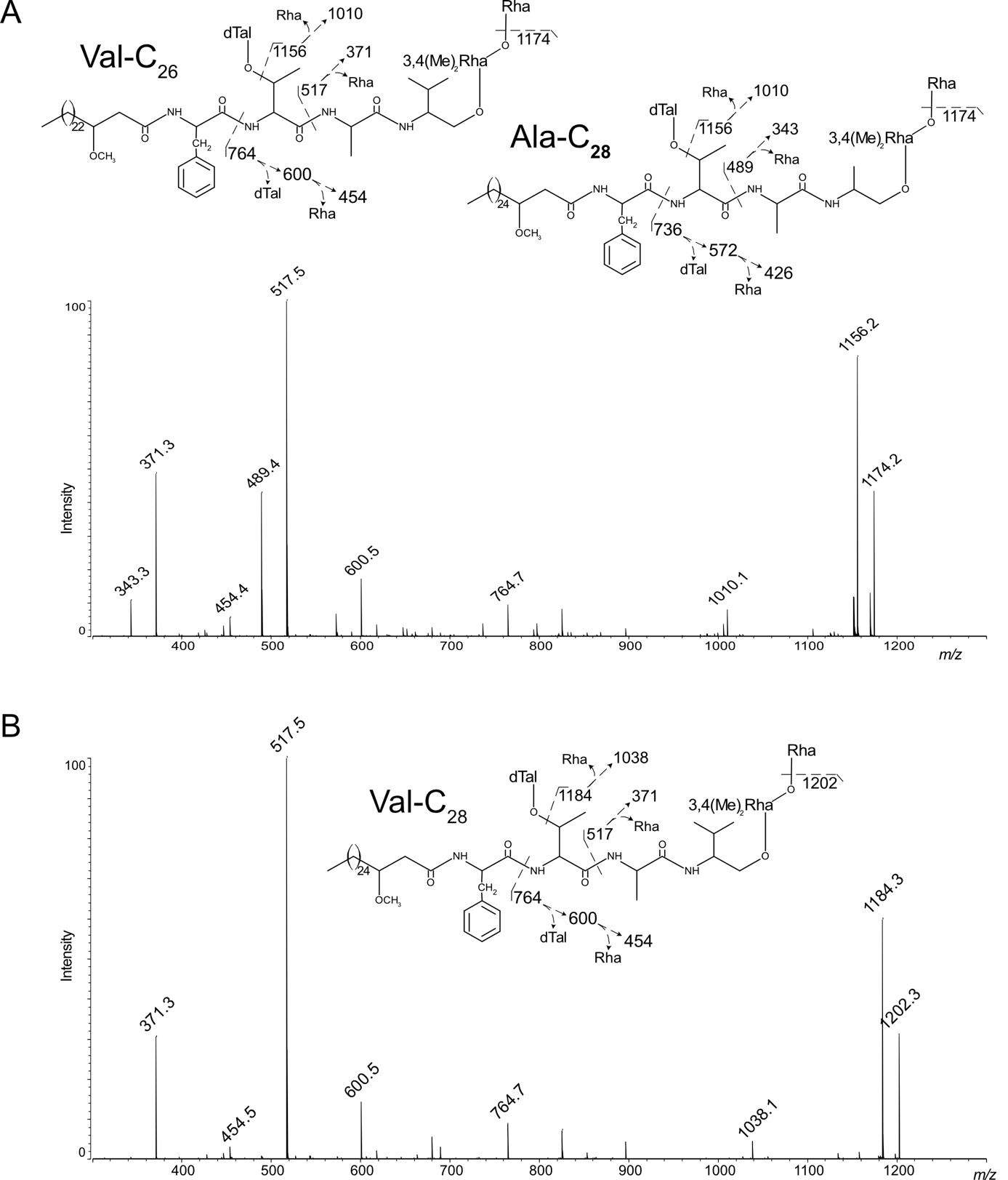


**Figure S6: (A)** MALDI-MS^2^ spectrum in positive mode of the selected parent ion at *m/z* 1320 of F1 purified fraction from Δ1,2,1725c,3448. Fragmentation patterns of two GPL-3 isomers Ala-C_28_ and Val-C_26_. **(B)** MALDI-MS^2^ spectrum in positive mode of the selected parent ion at *m/z* 1348 from Δ1,2,1725c,3448 mutant strain. Fragmentation pattern of GPL-3 Val-C_28_ is shown.


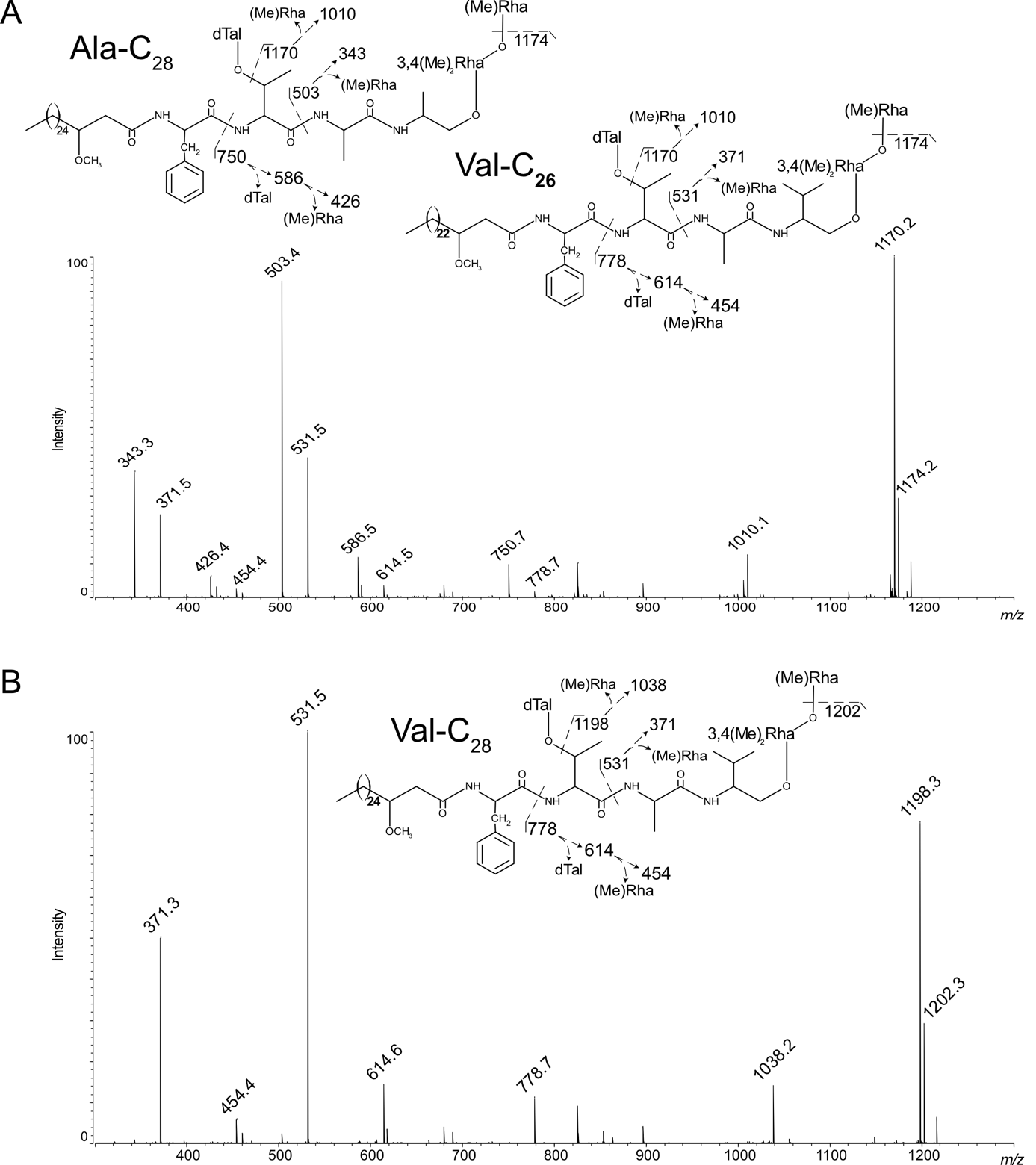


**Figure S7:** **(A)** MALDI-MS^2^ spectrum in positive mode of the selected parent ion at *m/z* 1334 of F2 purified fraction from Δ1,2,1725c,3448. Fragmentation patterns of two GPL-3 isomers Ala-C_28_ and Val-C_26_ are shown **(B)** MALDI-MS^2^ spectrum in positive mode of the selected parent ion at *m/z* 1362 of F2 purified fraction from Δ1,2,1725c,3448. Fragmentation pattern of GPL-3 Val-C_28_is shown.


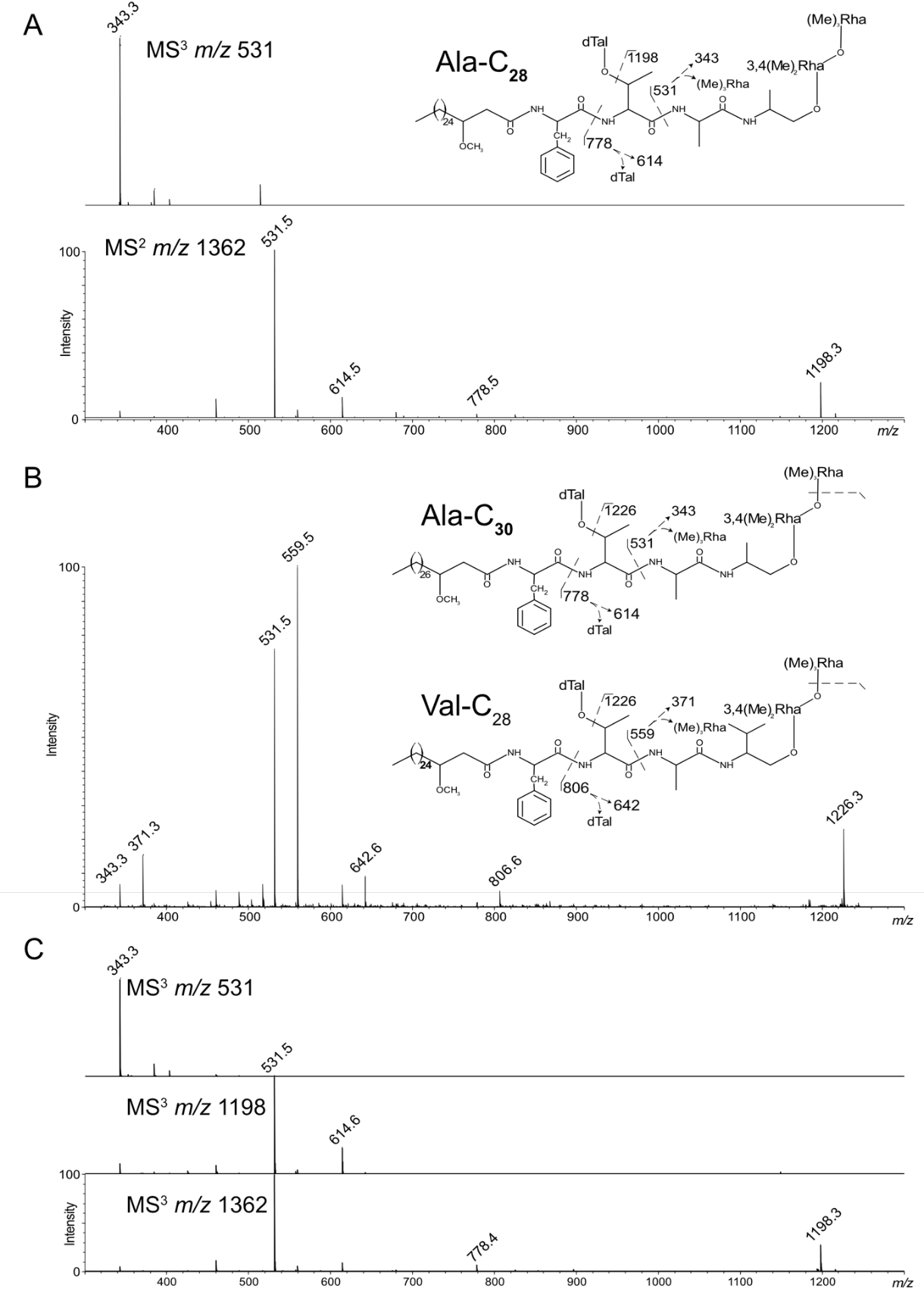


**Figure S8: (A)** MALDI-MS^2^ spectrum in positive mode of the selected parent ion at *m/z* 1362, superimposed with MS^3^ of fragment ion at *m/z* 531 of F3 purified fraction from Δ1,2,1725c,3448. Fragmentation pattern of GPL-3 Ala-C_28_ is shown. **(B)** MALDI-MS^2^ spectrum in positive mode of the selected parent ion at *m/z* 1390 of F3 purified fraction from Δ1,2,1725c,3448. Fragmentation patterns of two GPL-3 isomers Ala-C_30_ and Val-C_28_ are shown. **(C)** MALDI-MS^2^ spectrum in positive mode of the selected parent ion at *m/z* 1362, superimposed with MS^3^ of fragment ions at *m/z* 1198 and 531 of F4 purified fraction from Δ1,2,1725c. These two fractions exhibited similar spectra.


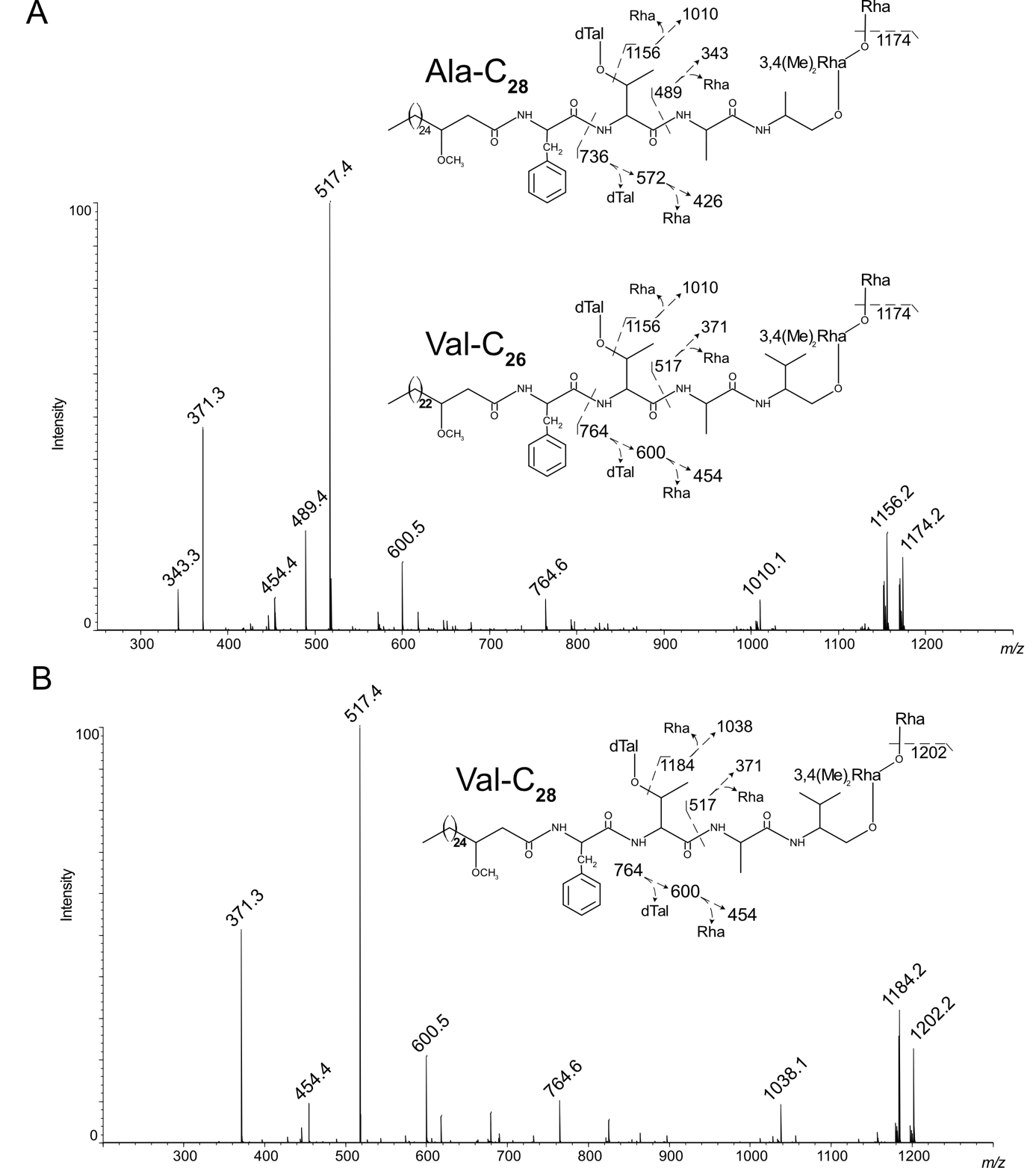


**Figure S9: (A)** MALDI-MS^2^ spectrum in positive mode of the selected parent ion at *m/z* 1320 of F1 purified fraction from Δ1,2,1725c. Fragmentation patterns of two GPL-3 isomers Ala-C_28_ and Val-C_26_ are shown. **(B)** MALDI-MS^2^ spectrum in positive mode of the selected parent ion at *m/z* 1348 of the same fraction. Fragmentation pattern of GPL-3 Val-C_28_is shown.


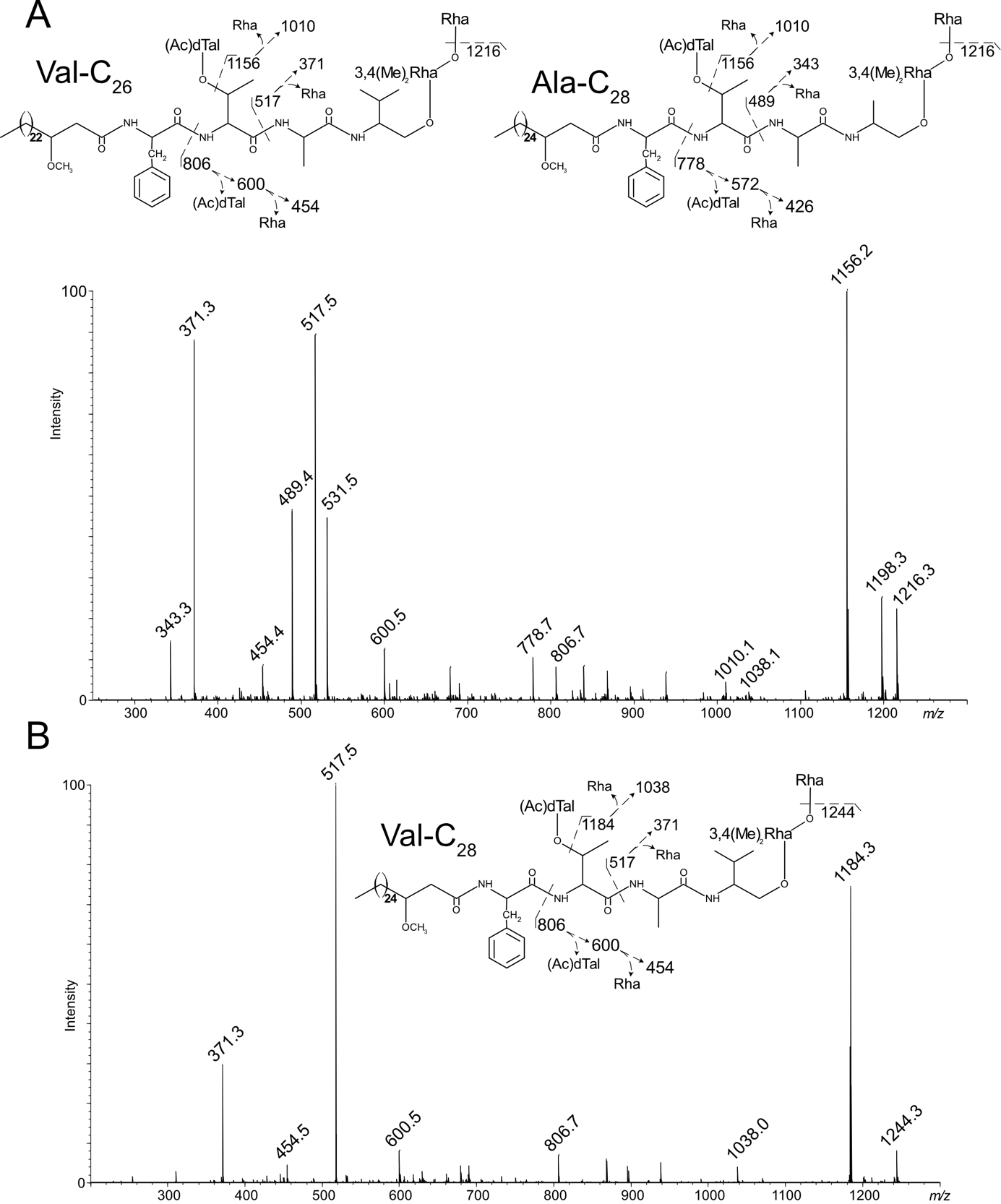


**Figure S10: (A)** MALDI-MS^2^ spectrum in positive mode of the selected parent ion at *m/z* 1362 of F2 purified fraction from Δ1,2, 1725c. Fragmentation pattern of two GPL-3 isomers are shown. **(B)** MALDI-MS^2^ spectrum in positive mode of the selected parent ion at *m/z* 1390 of the same fraction. Fragmentation pattern of mono-*O*-acetylated GPL-3 Val-C_28_.


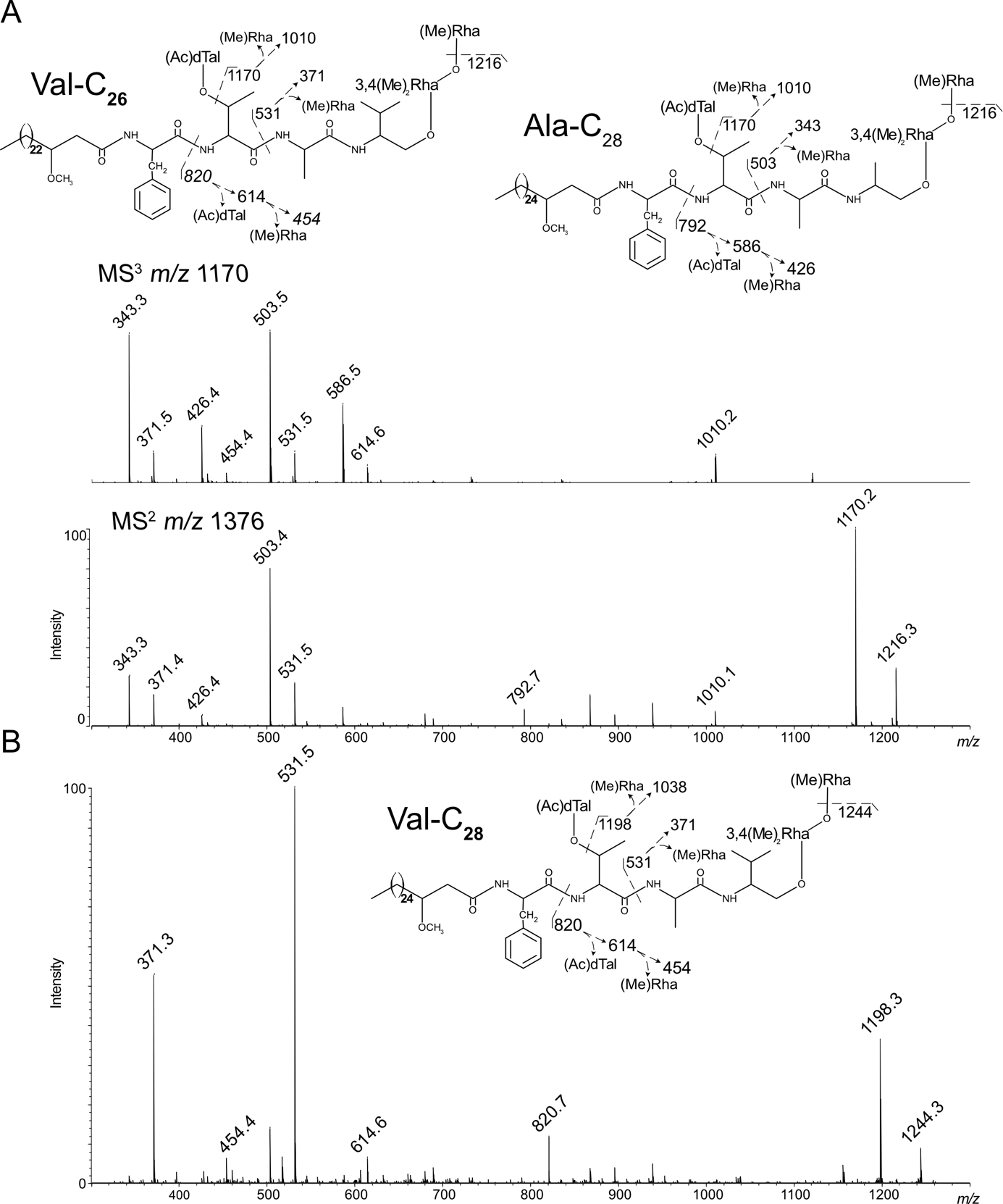


**Figure S11:** **(A)** MALDI-MS^2^ spectrum in positive mode of the selected parent ion at *m/z* 1376 of F3 purified fraction from Δ1,2,1725c, superimposed with MALDI-MS^3^ spectrum of fragment ion at *m/z* 1170, confirming the loss of mono-*O*-methyl Rha. Fragmentation patterns of two GPL-3 isomers Ala-C_28_ and Val-C_26_ are shown. **(B)** MALDI-MS^2^ spectrum in positive mode of the selected parent ion at *m/z* 1404 of the same fraction. Fragmentation pattern of GPL-3 Val-C_28_ is shown.


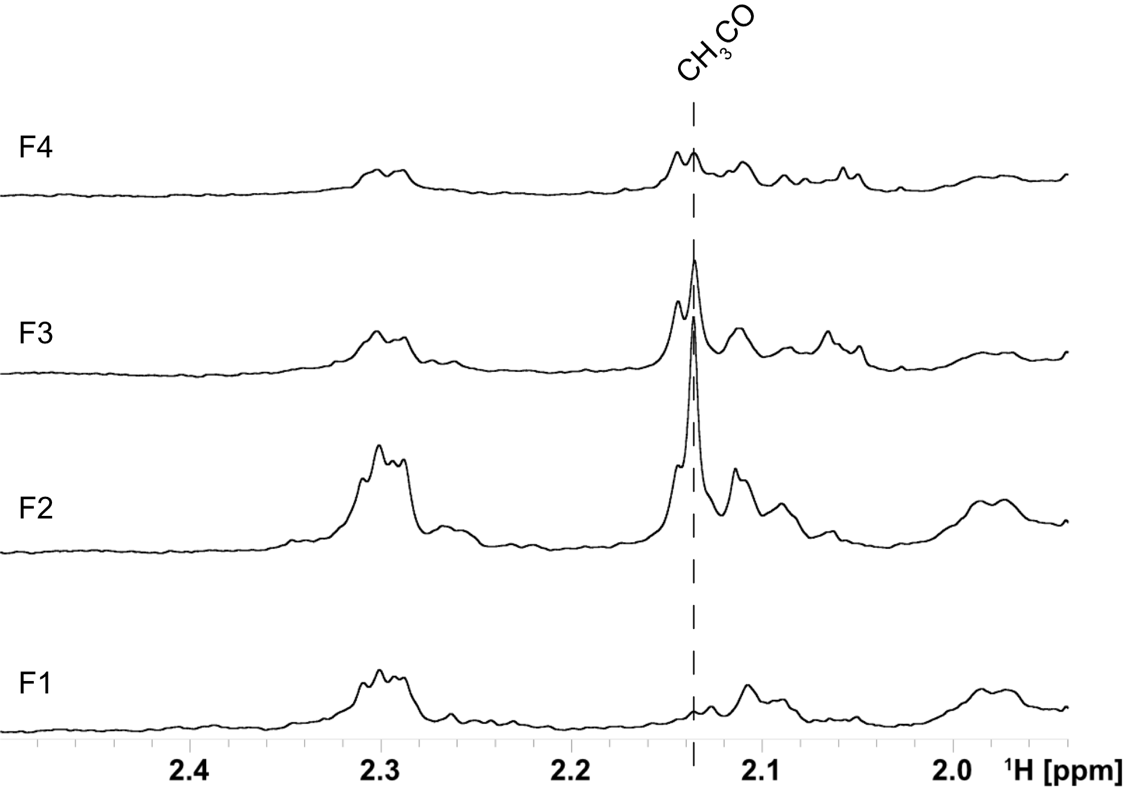


**Figure S12:** ^1^H NMR spectra of F1 to F4 purified fractions from Δ1,2,1725c that show a clear signal at δ2,14 ppm attributed to acetyl group in F2 and F3 fractions.


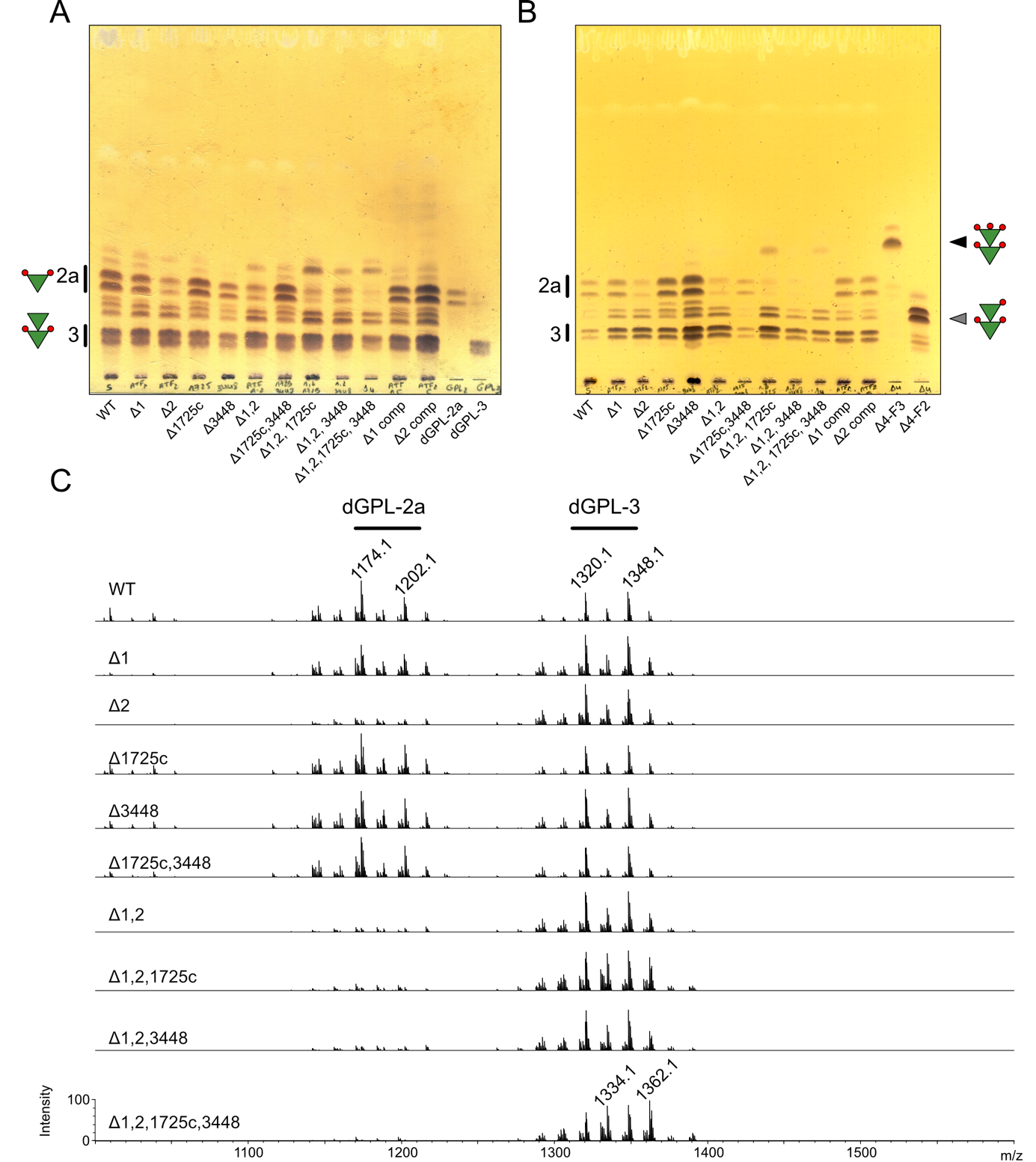


**Figure S13:** **(A, B)** TLC analysis of the saponified polar lipid fraction from WT and *atf* mutants. Butanol extracted GPLs were developed once with CHCl_3_/MeOH/H_2_0 (90:10:1, v/v/v) and stained with orcinol. Purified GPL-2a and GPL-3 were submitted to the same treatment **(A)** as well as F2 and F3 fractions from Δ1,2, 1725c,3448 **(B)**. **(C)** MALDI-MS spectra in positive mode of the saponified GPL from WT and *atf* mutants. Previously characterized de-*O*-acetylated GPL-2a (dGPL-2a) and GPL-3 (dGPL-3) are shown on top of the spectra (24).


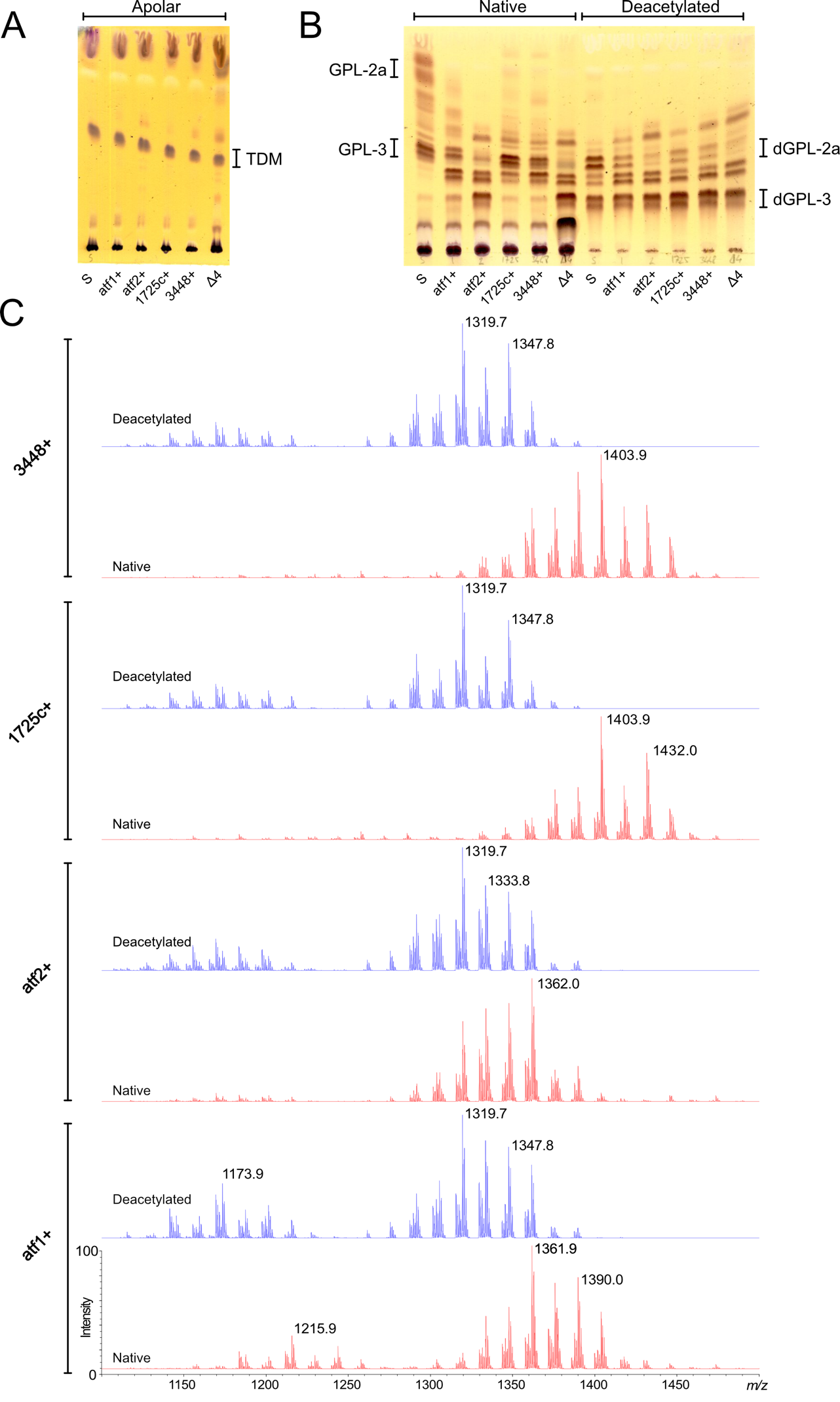


**Figure S14:** TLC analysis of the apolar lipid fraction **(A)** and the native or deacetylated polar lipid fraction **(B)** of WT and quadruple mutant strains individually expressing each acetyltransferase. Glycolipids or butanol-extracted GPLs were developed once with CHCl_3_/MeOH/H_2_0 (90:10:1, v/v/v) and stained with orcinol. **(C)** Positive mode MALDI-MS spectra of the native and deacetylated polar lipid fraction derived from WT and quadruple mutant strains individually expressing each acetyltransferase.

**Figure S15:** **RNAseq analysis of prophage-encoded *atf* genes**. Below is shown the left parts (~ 7 kbp) of the closely-related prophages prophiATCC19977-1 and prophiGD17-2, both members of Subcluster MabA1. The genes are shown as colored boxes above or below each genome maker, reflecting rightwards- and leftwards-transcription respectively. The purple shading between the genomes reflects BLASTN nucleotide sequence similarity, and the genomes are 100% identical in this segment. The genomes are aligned with the RNAseq data above for *M. abscessus* GD17, as reported previously (41), showing the numbers of reads mapping to the forward and reverse strands as indicated, from 0 to 100. The left ends of the prophages correspond to the left attachment site, *attL*, as indicated.

**SUPPLEMENTARY TABLE**

**Table S1:** Plasmids used in this study.

| Plasmids | | | |
| --- | --- | --- | --- |
| pTEC27 | *tdTomato* expressed under the control of a strong mycobacterial promoter by a multicopy *E. coli*/mycobacterial shuttle vector | Hyg | Addgene (plasmid 30182) |
| pUX1*-katG* | A pUX1 variant including *katG* gene of *M. tuberculosis* as a marker to counter-select in the presence of isoniazid and allowing to generate unmarked chromosomal alterations. | Kan, Hyg | (25) |
| pUX1-*katG*-*atf1* | pUX1-*katG* including the upstream and downstream sequences around *atf1* | Kan | This study |
| pUX1*-katG*-*atf2* | pUX1-*katG* including the upstream and downstream sequences around *atf2* | Kan | This study |
| pUX1-*katG*-*MAB-1725c* | pUX1-*katG* including the upstream and downstream sequences around *MAB-1725c* | Kan | This study |
| pUX1-*katG*-*MAB_3448* | pUX1-*katG* including the upstream and downstream sequences around *MAB-3448* | Kan | This study |
| pMV306 | Integrative vector | Kan | (38) |
| pMV306-*atf1* | pMV306 enabling the expression of Atf1-HA in mycobacteria | Kan | This study |
| pMV306-*atf2* | pMV306 enabling the expression of Atf2-HA in mycobacteria | Kan | This study |
| pMV306-*MAB_1725c* | pMV306 enabling the expression of MAB-1725c-HA in mycobacteria | Kan | This study |
| pMV306-*MAB_3448* | pMV306 enabling the expression of MAB-3448-HA in mycobacteria | Kan | This study |

Hyg, hygromycin; Kan, kanamycin

**Table S2:** Primers used in this study.

| **Number** | | | **5’→3’ sequence** | **Restriction site** | **F (forward),**  **R (reverse)** |
| --- | --- | --- | --- | --- | --- |
| **Cloning in pUX1-*katG*** | | | | | |
| 1 | Atf1 U (F) | | GAGA**TTAATTAA**AACACCACGCTGATGTCTGCGG | PacI | F |
| 2 | Atf1 U (R) | | GAGA**CAATTG**GAGTGTCAAACCCTTAGCCTGCC | MfeI | R |
| 3 | Atf1 D (F) | | GAGA**CAATTG**CCGATTCCTCTTGATCGCGATGCT | MfeI | F |
| 4 | Atf1 D (R) | | GAGA**GCTAGC**GGATAGTGTCACCAAGGCCGCCGA | NheI | R |
| 5 | Atf2 U (F) | | GAGA**TTAATTAA**CACAGCACAGTCGCTTTCGGAG | PacI | F |
| 6 | Atf2 U (R) | | GAGA**CAATTG**GTTCAGCGCGTTGTTTCGTGAATCGA | MfeI | R |
| 7 | Atf2 D (F) | | GAGA**CAATTG**CTGATCGAGAAACGCGCGCTG | MfeI | F |
| 8 | Atf2 D (R) | | GAGA**GCTAGC**GAATCACGACCACCGAGTCGAAC | NheI | R |
| 9 | MAB_1725c U (F) | | GAGA**TTAATTAA**ACTGCGATGGGAACCAGGTTCG | PacI | F |
| 10 | MAB_1725c U (R) | | GAGA**CAATTG**GAGCGCGTTGTTGCGTGGGT | MfeI | R |
| 11 | MAB_1725c D (F) | | GAGA**GAATTC**GAGAAACGCACTCTGGCGTTGAA | EcoRI | F |
| 12 | MAB_1725c D (R) | | GAGA**GCTAGC**TAGTGAGCATGATTGCCGTGCGTTG | NheI | R |
| 13 | MAB_3448 U (F) | | GAGA**TTAATTAA**ATGAGCTGATACCGAATCCACTGCTTG | PacI | F |
| 14 | MAB_3448 U (R) | | GAGA**CAATTG**TGCGTTAAGTGCGTTGTTACGCGGA | MfeI | R |
| 15 | MAB_3448 D (F) | | GAGA**CAATTG**GCACTCAAGAAACGGCTGCGC | MfeI | F |
| 16 | MAB_3448 D (R) | | GAGA**TCTAGA**GATCGTTGTCATGCTGGTCTATTTCGGA | XbaI | R |
| **Primers to verify double homologous recombination** | | | | | |
| 17 | | Atf1 | GACGTGTATCGCAATTTCTGGACGTTCTTCT | - | F |
| 18 | | Atf1 | GAAATCAACTTAGCCGACTCAAGGTAGGTGCT | - | R |
| 19 | | Atf2 | GATTCATTGGATCCGCGCTGTCCAA | - | F |
| 20 | | Atf2 | CTGCATTTTCGGTGGAATCTGTCAAAAAATCTTATG | - | R |
| 21 | | MAB_1725c | AACATACGGCAAACATCTGGGCATTACAAG | - | F |
| 22 | | MAB_1725c | TCTAACCTCAACGAGCTGCTGAGCTAA | - | R |
| 23 | | MAB_3448 | CGCTCGAAGCTCAATAGGCCAGTAA | - | F |
| 24 | | MAB_3448 | CTGCACTGGGCAGTGCTGTTGT | - | R |
| **Cloning in pMV306 under the *hsp60* promoter** | | | | | |
| 25 | | Atf1 | GAGA**CAATTG**CCATGACCAAGGCGGGTGCGACC | MfeI | F |
| 26 | | Atf1 | GAGA**GTTAAC**CTAAGCGTAATCTGGAACATCGTATGGGTACGCAGCATCGCGATCAAGAGGA | HpaI | R |
| 27 | | Atf2 | GAGA**GAATTC**CCATGAAGCTCGGTTCTGTATTCGATTCACGA | EcoRI | F |
| 28 | | Atf2 | GAGA**GTTAAC**CTAAGCGTAATCTGGAACATCGTATGGGTAACTGTCCTCATGCGGCGACAGT | HpaI | R |
| 29 | | MAB_1725c | GAGA**GAATTC**CCGTGCTCGGACGCGTATTCGAC | EcoRI | F |
| 30 | | MAB_1725c | GAGA**GTTAAC**CTAAGCGTAATCTGGAACATCGTATGGGTAGACGGCCACCACTTGCTTC | HpaI | R |
| 31 | | MAB_3448 | GAGA**GAATTC**CCGTGCTGGGACGGGTATTTGATCC | EcoRI | F |
| 32 | | MAB_3448 | GAGA**GTCGAC**CTAAGCGTAATCTGGAACATCGTATGGGTACCCCGCACCCGCTTTGACGCGCA | SalI | R |

**Table S3:** Strains used in this study.

| **Name** | **Description/genotype** | **Resistance** | **Reference** |
| --- | --- | --- | --- |
| *M. abscessus*  Smooth (S) | *M. abscessus* *sensu stricto*, strain CIP104536^T^, S morphotype | - | Laboratoire de Référence des Mycobactéries |
| *M. abscessus*  Rough (R) | *M. abscessus* *sensu stricto*, strain CIP104536^T^, R morphotype | - | Laboratoire de Référence des Mycobactéries |
| Δ*1* | Unmarked deletion of *atf1* (*MAB_4106c*) in *M. abscessus* | - | This study |
| Δ*2* | Unmarked deletion of *atf2* (*MAB_4110c*) in *M. abscessus* | - | This study |
| Δ*MAB_1725c* | Unmarked deletion of *MAB_1725c* in *M. abscessus* | - | This study |
| Δ*MAB_3448* | Unmarked deletion of *MAB_3448* in *M. abscessus* | - | This study |
| Δ*1, 2* | Unmarked deletion of *atf1*(*MAB_4106c*) and *atf2* (*MAB_4110c*) in *M. abscessus* | - | This study |
| Δ*MAB_1725c, MAB_3448* | Unmarked deletion of *MAB_1725c* and *MAB_3448* in *M. abscessus* | - | This study |
| Δ*1, 2, MAB_1725c* | Unmarked deletion of *atf1*, *atf2* and *MAB_1725c* in *M. abscessus* | - | This study |
| Δ*1, 2, MAB_3448* | Unmarked deletion of *atf1*, *atf2* and *MAB_3448* in *M. abscessus* | - | This study |
| Δ*4* | Unmarked deletion of *atf1*, *atf2*, *MAB_1725c* and *MAB_3448* in *M. abscessus* | - | This study |
| Δ*1* + pMV306-*atf1-HA* | Δ*atf1* carrying pMV306-*atf1* | KAN | This study |
| Δ*2* + pMV306-*atf2-HA* | Δ*atf2* carrying pMV306-*atf2* | KAN | This study |
| Δ*MAB_1725c* + pMV306-*MAB_1725c-HA*  Δ*MAB_3448* + pMV306-*MAB_3448-HA* | Δ*MAB_1725c* carrying pMV306-*MAB_1725c*  Δ*MAB_3448* carrying pMV306-*MAB_3448* | KAN  KAN | This study  This study |
| Δ*4* + pMV306-*atf1-HA*  Δ*4* + pMV306-*atf2-HA*  Δ*4* + pMV306-*MAB_1725c-HA*  Δ*4* + pMV306-*MAB_3448-HA* | Δ*4* carrying pMV306-*atf1*  Δ*4* carrying pMV306-*atf2*  Δ*4* carrying pMV306-*MAB_1725c*  Δ*4* carrying pMV306-*MAB_3448* | KAN  KAN  KAN  KAN | This study  This study  This study  This study |

**Table S4:** **Distribution of the different genes coding for acetyltransferases in selected GPL-producing mycobacteria.** The “+” sign indicates the presence of *atf* or an orthologue of *atf*. The “-” symbol denotes the absence of the indicated gene. Bioinformatics analysis was performed using KEGG (https://www.genome.jp/kegg/). The four *M. abscessus* genes were blasted using the ortholog function of the software.

**
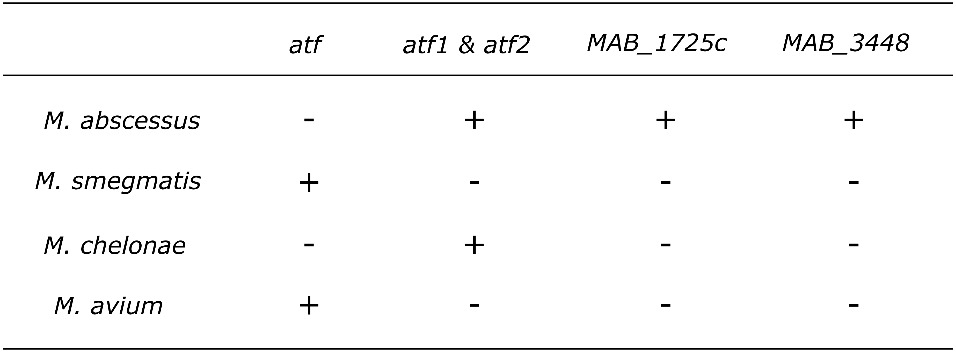
**
